# Supplementary material for: Trends, barriers and enablers to measles immunisation coverage in Saskatchewan, Canada: A mixed methods study
Source: PLoS One. 2022 Nov 23;17(11):e0277876. doi: 10.1371/journal.pone.0277876 (PMC9683619; doi:10.1371/journal.pone.0277876)
Supplement: S1 File — (DOCX) [file pone.0277876.s001.docx]

# Supporting Information

**Trends, Barriers and Enablers to Measles Immunisation Coverage in Saskatchewan, Canada: a Mixed Methods Study**

**Short title**: Measles immunisation coverage in Saskatchewan, Canada

Marcus M Ilesanmi^1^(ORCID: 0000-0003-2086-7923), Sylvia Abonyi^1,2^ (ORCID: 0000-0003-2657-4144), Punam Pahwa^1,3^(ORCID: 0000-0002-3342-0496), Volker Gerdts^4,5^(ORCID: 0000-0001-8229-1611) Michael Scwandt^6,7^(ORCID: 0000-0002-3164-8972), Cordell Neudorf^1,8^ (ORCID: 0000-0003-4943-4785)

^1^Department of Community Health and Epidemiology, College of Medicine, University of Saskatchewan, Saskatoon, SK, Canada.

^2^Saskatchewan Population Health and Evaluation Research Unit (SPHERU), University of Saskatchewan, SK, Canada

^3^Canadian Centre for Health and Safety in Agriculture, University of Saskatchewan, Saskatoon, SK, Canada

^4^Vaccine and Infectious Disease Organization-International Vaccine Centre (VIDO-InterVac), University of Saskatchewan, Saskatoon, SK, Canada

^5^Department of Veterinary Microbiology, Western College of Veterinary Medicine, University of Saskatchewan, Saskatoon, SK, Canada

^6^University of British Columbia, School of Population and Public Health, Vancouver, BC, Canada

^7^Vancouver Coastal Health, Office of the Chief Medical Health Officer, Vancouver, BC, Canada

^8^Health Surveillance & Reporting, Saskatchewan Health Authority (SHA), Saskatoon, SK, Canada

***Corresponding Author:**

E-mail: [marcus.ilesanmi@usask.ca](mailto:marcus.ilesanmi@usask.ca) (MMI)

## Appendix 1:

## First phase Qualitative Strand Interview Guide (Pre data analysis)

### Questions/Interview Guide

**Introduction:**

- How long have you been at your position?

**Questions**

- What is your role in the measles immunization program in your health region?
- What has measles immunization coverage been for your health region in the past?
- What is the current level of priority for improving measles immunization coverage among 0 – 2-year-old children in your RHA?
- What targets for immunization coverage would you propose for your RHA for the next two years?
  - What do you think are the barriers to achieving a higher coverage rate in your RHA?

In your RHA;

- - What strategies have you used to increase the uptake of measles immunizations?
  - Did this work equally well with all populations (groups)?
    - Were there populations that you feel were not responsive to these strategies?
    - What might work better?
  - What can you sustain personally or as a health region?
    - Provide reasons for challenges with sustainability.
  - What are the strengths you think you can build on?
    - What initiatives are needed in your RHA to ensure continued improvement in coverage rates
  - How have you engaged patients/families, providers, the media, the public on awareness of the need for immunization or the barriers to immunization? What role does the media play?
    - What are some of the challenges encountered?

## Appendix 2:

## Second phase Qualitative Strand Interview Guide (After Immunisation data analysis and presentation of initial findings)

### Questions/Interview Guide

- Has your role in the measles immunization program in your health region changed since the last interview?
- Now that we have seen the analyzed results, from your perspective as a healthcare provider, what more would you like to do to improve immunization coverage figures in your area?
- What innovations in the interventions will you carry out to improve equitable measles immunization coverage rates in your health region/area?
  - Is this intervention universal or targeted?
  - Did you make adjustments for some sub-groups?
- What do you expect the institution you represent to do differently, going forward?
