# Supplementary material for: Trends, barriers and enablers to measles immunisation coverage in Saskatchewan, Canada: A mixed methods study
Source: PLoS One. 2022 Nov 23;17(11):e0277876. doi: 10.1371/journal.pone.0277876 (PMC9683619; doi:10.1371/journal.pone.0277876)

**Supporting information S3.** **Saskatchewan Geographical Distribution by proportion of <2year children (2002 – 2013)**


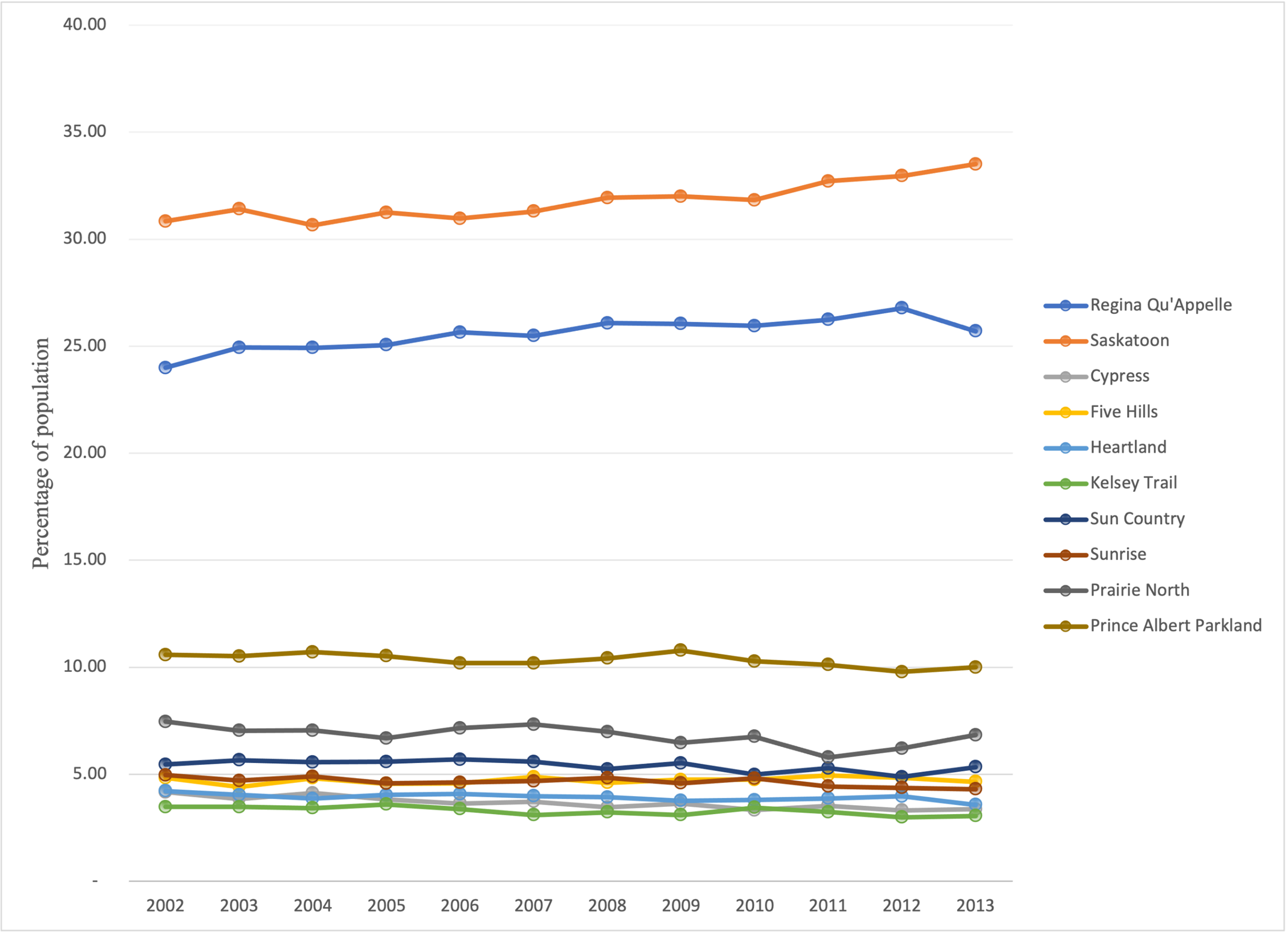

Supplement: S1 Fig — (DOCX) [file pone.0277876.s002.docx]
