# Supplementary material for: Trends, barriers and enablers to measles immunisation coverage in Saskatchewan, Canada: A mixed methods study
Source: PLoS One. 2022 Nov 23;17(11):e0277876. doi: 10.1371/journal.pone.0277876 (PMC9683619; doi:10.1371/journal.pone.0277876)
Supplement: Attachment — Submitted filename: PONE-D-21-15652_R1_reviewed_81922.pdf [file pone.0277876.s004.pdf]

PLOS ONE

Trends, Barriers and Enablers to Measles Immunization Coverage in Saskatchewan, Canada: a Mixed Methods Study

--Manuscript Draft--

**Manuscript Number:**	PONE-D-21-15652R1
**Article Type:**	Research Article
**Full Title:**	Trends, Barriers and Enablers to Measles Immunization Coverage in Saskatchewan, Canada: a Mixed Methods Study
**Short Title:**	Measles immunization coverage in Saskatchewan, Canada
**Corresponding Author:**	Marcus M Ilesanmi, MB.BS., Ph.D<br>University of Saskatchewan<br>Saskatoon, Saskatchewan CANADA
**Keywords:**	measles; Immunisation coverage; Coverage disparity; Health providers perspective; equity; Joinpoint regression
**Abstract:**	Many social, cultural, and systemic challenges affect the uptake of measles immunisation services. Prior studies have looked at the caregivers' perspectives, but little is known about the perspectives of the health care providers on the barriers of measles immunisation services in Canada. This study examined measles immunisation coverage trends across the regional health authorities in Saskatchewan and explored the barriers and enablers to measles immunisation coverage from providers' perspectives. The study adopted an explanatory sequential mixed method. We utilized the entire population of 16,582 children under two years of age available in the Saskatchewan Immunisation Management System (SIMS) registry for 2002 and 2013 in aggregate format and interviewed 18 key informants in pre-determined two-stages in 2016 and 2017. The quantitative analysis was done with Joinpoint regression modelling, while the qualitative interview data was analyzed using hybrid inductive and deductive thematic approaches.<br>There was a 16.89%-point increase in measles immunisation coverage in the province from 56.32% to 73.21% between 2002 and 2013. There was also a persistently higher coverage among the affluent (66.95% - 82.37%) than the most deprived individuals (45.79% - 62.60%) in the study period. The annual rate of coverage change was marginally higher among the most deprived (16.81%; and average annual percentage change (AAPC) 2.0, 95% CI 1.7 – 2.2) than among the affluent group (15.42% and AAPC 3.0; 95% CI 2.0 – 4.0). While access-related issues, caregivers' fears, hesitancy, anti-vaccination challenges, and resource limitations were barriers to immunisation, improving community engagement, service delivery flexibility, targeted social responses and increasing media role were found useful to address the uptake of measles and other vaccine-preventable diseases immunisation challenges.<br>There is low coverage and inequity in measles immunisation uptake in Saskatchewan from social and institutional barriers. Even though there is evidence of disparity reduction among the different groups, the barriers to increasing measles immunisation coverage have implications for the health of the socio-economically deprived groups, the healthcare system and other vaccination programs. There is a need to improve policy framework for community engagement, targeted programs, and public health discourse.
**Order of Authors:**	Marcus M Ilesanmi, MB.BS., Ph.D
	Sylvia Abonyi
	Punam Pahwa
	Volker Gerdts
	Michael Scwandt
	Cordell Neudorf
**Response to Reviewers:**	Authors' response: Trends, Barriers and Enablers to Measles Immunization Coverage in Saskatchewan, Canada: a Mixed Methods Study

Powered by Editorial Manager® and ProduXion Manager® from Aries Systems Corporation

Reviewer #1: General comment
R1.1: The topic of the article proposed by the researchers is interesting, it is a contribution to the analysis of measles vaccination trends, from a mixed approach. The data is limited to the province of Saskatchewan but could be understood given the qualitative approach the authors add to the study.
Authors response: Thank you for your constructive comments
Changes made: None

R1.2: However, I am concerned about the lack of concurrency in the temporality of the quantitative and qualitative approaches. I can understand that the quantitative data for this study only includes up to the year 2013, while the qualitative analysis would correspond to a more current information collection (the authors do not report on this). If so, this temporal gap would mean that the social, historical and cultural context in which the analysis is carried out is not the same. Ideally, I would suggest to the authors to extend the trend analysis to a more current date, closer to the period in which the data from the qualitative approach were collected.
Authors response: We looked more into contextuality in this study. The study looked at data from 2002 to 2013 which were the available data in the Saskatchewan Immunization registry at the time of the study. We intentionally used data to make the key informants (mostly experienced managers) aware of the gap and or challenges in measles immunization coverage to have them pursue vigorously quality and equity initiatives. The first interview with the key informants took place in the third quarter of 2016 at which time the only available data on the SIMS registry were updated until 2013 and the interviews were with people who had been working in the system for many years and they reflected over that history and brought to fore what they have done in that period. The information pertaining to their work in those periods when the data were collected were asked. The information acquired were aimed at informing future interventions and practice. We conducted the first interview when they had not seen the findings from the quantitative analysis. The coverage rates that the RHAs had seen before our study was the overall coverage for their respective RHAs as made available by the Ministry of Health in a non-granular form like our study did. Our study looked at rural-urban disparity in coverages as well as different socio-economic groups. We then collated the generalized themes to see what were done in different RHAs and compared that with the outcome in terms of coverage. Subsequently, we shared the results of the quantitative analysis with the same group of interviewees to see if they have made improvements or not especially with new evidence and the new details on geographical and socio-economic analysis. With that level of analysis, we carried out knowledge translation with the informants. This led to the second stage interviews in 2018, when we interrogated the same set of people about the past and reflect on how they wanted to use the new information to make future programmatic changes. We think their responses reflected not just that point in time but their experience over the last decade and their future plans, therefore, there is no substantial gap that could have affected the results.
Changes made: Page 7, line 168 – 174: Eighty-three percent (n=15) of the interview participants had at least eleven years' experience, either in their present positions or in Saskatchewan RHAs in various public and population health capacities; with MHOs, an average of thirteen years' experience and Coordinators/Front-line Immunization Officers, an average of twenty years. The information asked from the participants pertained to their work in those periods when the quantitative data were collected; hence they were reflecting on their experience during the time data were collected, taking into account the past and future implications.
Also, page 23, lines 563 – 567: However, the 2016 and 2017 qualitative data provided by key informants, majority of who were long serving in the RHAs were valuable in providing a comprehensive picture of trends and challenges faced in their respective RHAs addressing both interventions in the retrospective periods dating to a decade and how the new information will be used to make changes in the future.

Summary
R1.3: Line 32. It is not clear in what time or periods of time the interviews with the key informants were taken.
Authors response: We have added the time of the interview
Changes made: Page 2, Lines 30 – 33: We utilized the entire population of 16,582

Powered by Editorial Manager® and ProduXion Manager® from Aries Systems Corporation

children under two years of age available in the Saskatchewan Immunization Management System (SIMS) registry for 2002 and 2013 in aggregate format and interviewed 18 key informants in pre-determined two-stages in 2016 and 2017.

Introduction
R1.4: Lines 75-78. Specify in the province of Saskatchewan, what is the regular schedule of measles vaccination in the first and second doses.
Authors' response: The routine schedule for first and second measles vaccination has been specified.
Change made: Page 3, lines 73 – 75: The Saskatchewan routine measles immunisation schedule are two doses of measles containing vaccine with first dose recommended at one year (12 months) and a second dose starting from 18 months of age.[1]

Materials and methods
R1.5: Line 145. Specify if the vaccination coverage was obtained: annually? For the entire province or for each of the regions?
Authors' response: As recommended, we have added "annualized"
Changes made: Page 6, line 151 – 152: We used annualized data from two population groups, aged 12-14 months and 18-24 months.

R1.6: Justify why a data analysis is performed only up to 2013, why more recent data is not included.
Authors' response: This study took place at a period when health system transformation was taking place hence, we used all the available data up to that point.
Change made: Page 6, lines 145 – 149: This study took place at a period when health system transformation was taking place (i.e., from Saskatchewan Immunization Registry to the Panorama Gateway). The only available data at the time of study were up to 2013, hence the study used the total sample available in the province of Saskatchewan Immunization Management Registry.

R1.7: Lines 153-156. Describe in what time period the key informant interviews were conducted.
Authors' response: The time period has been specified.
Change made: Page 7, lines 159 – 164: All the selected eighteen key informants participated in the first phase of interviews while seventeen participated in the second phase. The first stage of interviews which took place in the fourth quarter of 2016 collected the interviewees' initial general perspectives on factors that promote or hinder measles immunisation uptake in their health regions while the second stage, in the fourth quarter of 2017, was conducted to validate key themes and insights from the initial interviews and after quantitative results were presented to the participants

R1.8: Describe whether there was triangulation of qualitative information and how it was done.
Authors' response: To ensure consistency in information received from each of the health regions, we interviewed two key informants.
Change made: Page 7, line 165 – 166: Qualitative data triangulation was ensured by interviewing two stakeholders each from each regional health authority.

R1.9: How do the authors justify a quantitative analysis of data from 2010-2013, while the qualitative analysis corresponds to a current period of time? That is, although there is a mixed approach to the research, the authors have worked with a quantitative and qualitative approach at two different, non-concurrent moments.
Authors' response: See response above (R1.2)
Change made: Response R1.2 above

Statistical analysis
R1.10: An analysis of differences by socioeconomic quintiles is carried out in the results. It is necessary to describe in the Statistical analysis section where this information was obtained and how it was included in the analysis.
Authors' response: We have described how socioeconomic quintiles were estimated in

	the methods section<br>Changes made: Page 9, lines 212 – 221: To assess socio-economic disparities, we used the index of deprivation developed at the Institut National de Sante Publique du Quebec (INSPQ) which measures deprivation at the level of dissemination areas (DAs), the smallest areas for which Census data are available in Canada, comprising of approximately 400 to 700 residents living in the same small area geography[50]. The deprivation index looked at social and material dimensions with the proportion of single parents, the proportion of residents living alone, and marital status in the social components while the material deprivation measured educational attainment, average income, and employment status variables.[50] The DAs were divided into five quintiles 1 to 5 (Q1 to Q5) where each quintile represented 20% of population with Q1 being the most privileged and Q5, the least. We referred to these quintiles as Socio-economic deprivation quintiles (S-EDQ1 – S-EDQ5) in this paper.<br><br>R1.11: I consider that, when evaluating the disparity in measles coverage by socioeconomic status, the authors could delve deeper into the results, there are some inequalities analyzes that could perhaps be applied in the present study, as well as exploring differences by urban, rural, between migrants, between indigenous and non-indigenous populations<br>Authors' response: Your suggestions are well noted. We have included information on urban and rural analysis in the results section as well as in the discussion. However, immunization data by ethnicity and immigration status were not systematically collected in the province of Saskatchewan. Also, the authors did not have access to individual but aggregated data for the study. We have hence noted this as part of study limitations.<br>Changes made: Pages 11 – 12, lines 268 – 278:<br>Assessing disparity in measles coverage by geographical location<br>There was increase in the coverage trends in Saskatchewan rural and urban locations with coverage rates being higher in the rural than in the urban locations. The coverage rate among on-time for second dose at 2-year (MCV2) increased between 2002 and 2008 (APC: 0.8, 95%CI:), followed by a higher progressive increase from 2008 till 2013 (APC: 3.0, 95%CI:) for the rural locations. . The urban location coverage rates displayed an increase from 2002 – 2008 at an APC of 2.3, a further increase of APC of 4.5 from 2008 – 2011 with the rate of change being significant with CIs of 1.7 – 3.0 and 1.6 – 7.5 respectively while the increase from 2011 – 2013 and an APC of 2.0, was not significant (CI -0.4 – 4.6). A test for parallelism of the two coverage rate trends for the on-time for MCV2 in the urban and rural locations to check for any difference between the two trend lines indicated no parallelism (p-value = 0.027) indicating a significant difference between the trend lines of the two geographical locations.<br>Page 13, lines 301 – 305: Summarizing, there was progressive increase in the coverage rates for both S-EDQ 1 and 5 and among both rural and urban populations for MCV2 in the province of Saskatchewan. The coverage rates were higher in the S-EDQ1 than S-EDQ5 and among the rural dwellers than the urban inhabitants among the on-time for MCV2 all through 2002 – 2013.<br>Page 24, line 568 – 570: It should be noted that data were not available for ethnicity and immigration status. Future studies are needed to explore patterns of measles immunization among other sub-populations which frequently exhibit inequities such as immigrants and indigenous population.<br><br>Results<br><br>R1.12: Lines 223-224. Are you referring to first or second dose coverage for measles, or full schedule coverage?<br>Authors' response: We have included that second dose for measles (MCV2) was used which is also the full schedule according to World Health Organisation (WHO).<br>Change made: Page 11, line 250 – 251: From MCV2 results for 2002, Sun Country RHA had the highest coverage of 67.22% while Prairie North RHA had the lowest at 49.15%.<br><br>R1.13: Line 240-251. Specify what S-EDQ1 means. It is the first time that the abbreviation has been used but I cannot find what it corresponds to in the text. In addition to mentioning it in the Abbreviations section, it should also be included in the

Powered by Editorial Manager® and ProduXion Manager® from Aries Systems Corporation

main text.
Authors’ response: In the edited document, the S-EDQ was first used on page 9, line 220 and the acronym has been fully specified.
Changes made: Page 9, line 220: We referred to these quintiles as Socio-economic deprivation quintiles (S-EDQ1 – S-EDQ5) in this paper.

R1.14: Again, I would suggest including additional analysis on inequality in vaccination coverage, for example, differences by urban and rural sector, between migrants, between indigenous and non-indigenous populations. Particularly, in the qualitative part, the differences between the urban and rural sectors are mentioned; It would be interesting to see if these differences are evident in the quantitative part.
Authors’ response: We have included a section on rural and urban sector in the quantitative results section.
Change made: Refer to R1.11 above.

Discussion

R.1.15: Line 430. What does HIT mean?
Authors’ response: HIT is an acronym for Herd Immunity Threshold. It was first mentioned in line 63. We have included the meaning of the acronym.
Change made: Page 3, line 61 – 63: Despite all the efforts to improve measles immunisation coverage, the proportion of immunised population with measles antigen containing vaccine (MCV) remains under the 95 percent herd immunity threshold (HIT).

R1.16: Line 433. To what period of time do these contextual adaptations refer (between 2002-2013? Or later).
Author’s response: The contextual adaptations period spanned from 2002 to 2013, employed currently and in the future depending on needs.
Changes made: Page 20, lines 487 – 490: All these efforts are towards tailoring immunisation service to the targeted beneficiaries [63]. These approaches were employed in different combinations by RHAs in the past, currently and into the future with modifications depending on contexts.

R1.17: Since when did promoters of community programs begin to be introduced? Could it be observed if the presence of community program promoters really had an effect on vaccination coverage?
Authors’ response: Community program promoters were first introduced in 2007 to address the inequities in vaccination coverage from the inner-city core neighborhoods. It was effective in improving the coverage even though it was capital intensive.
Change made: Page 20, lines 470 – 473: Particularly, regional health authorities where targeted approaches were introduced observed improvement in immunisation uptake by the initiation and maintenance of community program builders recruited from the community to reach out to the parents and clients in the inner-city core neighborhoods in Saskatoon RHA in 2007.
Page 20, lines 479 – 481: The above efforts are part of the strategies and interventions that increased uptake of measles immunisation especially by bridging the gap in the socio-economic divide with a resultant improvement in the coverage rates over the period under study.

R1.18: Line 448. What period of time do you refer to?
Authors’ response: We referred to the targeted approach to immunization period which started in Saskatchewan in 2007
Changes made: Page 20, lines 482 – 486: During the targeted approach period starting from 2007, many regional health authorities in the province of Saskatchewan utilized reminder systems more effectively, increased the number of immunisation service delivery points and became more flexible in terms of clinic opening hours to allow access for caregivers who could not attend within the strict regular opening hours

R1.19: Line 454 and following. I suggest, for the reader's clarity, to make a summary table of the changes over time in health strategies and policies related to vaccination, which are mentioned both in the results and in the discussion.

Powered by Editorial Manager® and ProduXion Manager® from Aries Systems Corporation

	Authors' response: The changes made varied in the respective RHAs. We have summarized the outcomes of the changes under the different themes in Fig 7 on page 19 of the manuscript.<br>Changes made: None<br><br><br>Figures.<br><br>R1.20: Figure 2. Include a note at the bottom of the figure that explains the graph and legends. Explain what S-EDQ is.<br>Author's response: The legend can be found on the figures.<br>Changes made: None<br><br><br>R1.21: Figure 5. Include a footnote to the figure that explains the graph and captions. Explain what is DEP 1, 2 etc.<br>Author's response: Footnote has been included. The DEP 1, 2 etc. are the same as S-EDQ 1, 2 etc. For uniformity and consistency, we have changed the DEP to S-EDQ on the graph.<br>Changes made: As indicated in the authors' response above.<br><br>R1.22: Figure 6. Include title, explain the main elements of the figure in a footnote.<br>Author's response: The title is indicated under the figure in the main document. The main elements were described in the main document. Figure 6 has become Figure 7 because of the inclusion of additional graph on rural and urban differences under results as part of the authors' response to R1.11.<br>Changes made: Page 18: lines 441 – 447: Figure 7 summarizes the results with various issues and clusters representing key building blocks—individual, social and institutional—that should be considered towards optimizing immunisation in the province. Each of these issues has critical implications for equity and public health policy. The framework includes individual, social and institutional dimensions of existing barriers and enablers and suggests a holistic, interconnected approach that integrates issues such as timeliness of measles immunisation dosages, population growth, urban-rural dichotomy, and regional differences and disparities in coverage rates.
**Additional Information:**	
**Question**	**Response**
**Financial Disclosure**<br><br>Enter a financial disclosure statement that describes the sources of funding for the work included in this submission. Review the submission guidelines for detailed requirements. View published research articles from *PLOS ONE* for specific examples.<br><br>This statement is required for submission and **will appear in the published article** if the submission is accepted. Please make sure it is accurate.	The author(s) received no specific funding for this work.

**Unfunded studies**<br>Enter: *The author(s) received no specific funding for this work.*<br><br>**Funded studies**<br>Enter a statement with the following details:<br>• Initials of the authors who received each award<br>• Grant numbers awarded to each author<br>• The full name of each funder<br>• URL of each funder website<br>• Did the sponsors or funders play any role in the study design, data collection and analysis, decision to publish, or preparation of the manuscript?<br>• **NO** - Include this sentence at the end of your statement: *The funders had no role in study design, data collection and analysis, decision to publish, or preparation of the manuscript.*<br>• **YES** - Specify the role(s) played.<br><br>* typeset	
**Competing Interests**<br><br>Use the instructions below to enter a competing interest statement for this submission. On behalf of all authors, disclose any competing interests that could be perceived to bias this work—acknowledging all financial support and any other relevant financial or non-financial competing interests.<br><br>This statement is required for submission and **will appear in the published article** if the submission is accepted. Please make sure it is accurate and that any funding sources listed in your Funding Information later in the submission form are also declared in your Financial Disclosure statement.<br><br>View published research articles from *PLOS ONE* for specific examples.	The authors have declared that no competing interests exist.

**NO authors have competing interests**<br><br>Enter: *The authors have declared that no competing interests exist.*<br><br>**Authors with competing interests**<br><br>Enter competing interest details beginning with this statement:<br><br>*I have read the journal's policy and the authors of this manuscript have the following competing interests: [insert competing interests here]*<br><br>* typeset	
**Ethics Statement**<br><br>Enter an ethics statement for this submission. This statement is required if the study involved:<br><br>• Human participants<br>• Human specimens or tissue<br>• Vertebrate animals or cephalopods<br>• Vertebrate embryos or tissues<br>• Field research<br><br>Write "N/A" if the submission does not require an ethics statement.<br><br>General guidance is provided below. Consult the submission guidelines for detailed instructions. **Make sure that all information entered here is included in the Methods section of the manuscript.**	This study was approved by the research ethics board of the University of Saskatchewan, Canada (BIO-16-18), Regina Qu'Appelle Health Region Research Ethics Board (REB-16-91) and all the remaining 9 participating Regional Health Authorities of the province of Saskatchewan. The immunisation data were extracted with researcher-generated syntax from the Saskatchewan Immunisation Database through Saskatchewan eHealth in a special format to ensure that the data remained anonymized and de-identified throughout the research process and collated through a provincial PHO leveraging upon the province data sharing agreement. All key informants had both written and verbal informed consents before interviews.

Powered by Editorial Manager® and ProduXion Manager® from Aries Systems Corporation

**Format for specific study types**<br><br>**Human Subject Research (involving human participants and/or tissue)**<br>• Give the name of the institutional review board or ethics committee that approved the study<br>• Include the approval number and/or a statement indicating approval of this research<br>• Indicate the form of consent obtained (written/oral) or the reason that consent was not obtained (e.g. the data were analyzed anonymously)<br><br>**Animal Research (involving vertebrate animals, embryos or tissues)**<br>• Provide the name of the Institutional Animal Care and Use Committee (IACUC) or other relevant ethics board that reviewed the study protocol, and indicate whether they approved this research or granted a formal waiver of ethical approval<br>• Include an approval number if one was obtained<br>• If the study involved *non-human primates*, add *additional details* about animal welfare and steps taken to ameliorate suffering<br>• If anesthesia, euthanasia, or any kind of animal sacrifice is part of the study, include briefly which substances and/or methods were applied<br><br>**Field Research**<br><br>Include the following details if this study involves the collection of plant, animal, or other materials from a natural setting:<br>• Field permit number<br>• Name of the institution or relevant body that granted permission	
**Data Availability**<br><br>Authors are required to make all data underlying the findings described fully available, without restriction, and from the time of publication. PLOS allows rare exceptions to address legal and ethical concerns. See the PLOS Data Policy and FAQ for detailed information.	No - some restrictions will apply

<table>
<tr><td>A Data Availability Statement describing where the data can be found is required at submission. Your answers to this question constitute the Data Availability Statement and will be published in the article, if accepted.

Important: Stating 'data available on request from the author' is not sufficient. If your data are only available upon request, select 'No' for the first question and explain your exceptional situation in the text box.

Do the authors confirm that all data underlying the findings described in their manuscript are fully available without restriction?</td><td></td></tr>
<tr><td>Describe where the data may be found in full sentences. If you are copying our sample text, replace any instances of XXX with the appropriate details.

• If the data are held or will be held in a public repository, include URLs, accession numbers or DOIs. If this information will only be available after acceptance, indicate this by ticking the box below. For example: All XXX files are available from the XXX database (accession number(s) XXX, XXX.).
• If the data are all contained within the manuscript and/or Supporting Information files, enter the following: All relevant data are within the manuscript and its Supporting Information files.
• If neither of these applies but you are able to provide details of access elsewhere, with or without limitations, please do so. For example:

Data cannot be shared publicly because of [XXX]. Data are available from the XXX Institutional Data Access / Ethics Committee (contact via XXX) for researchers who meet the criteria for access to confidential data.

The data underlying the results presented in the study are available from (include the name of the third party</td><td>Data cannot be shared publicly because of confidential issues. The data set used for the study is owned and in the custody of a third-party organization – the Saskatchewan provincial government. There are ethical restrictions on sharing the data of the Ministry of Health. Researchers who wish to carry out similar studies are expected to apply to the provincial Ministry of Health (Population Health Branch) for approval as the legal custodian of the immunization registry data which is currently hosted in Panorama.

For intending researchers, the data may be requested through:
https://www.ehealthsask.ca/health-data/analytics/Pages/Researcher-Access-to-Data.aspx

The requisition form can be found at the link:
http://www.ehealthsask.ca/forms/Forms/RequestForInformationForm.pdf

while the requisition should be mailed to: servicedesk@ehealthsask.ca

No special privileges were received by the authors in accessing the data from the third-party organization that other researchers would not have.</td></tr>
</table>

and contact information or URL).<br>• This text is appropriate if the data are owned by a third party and authors do not have permission to share the data.<br><br>* typeset	
Additional data availability information:	

Powered by Editorial Manager® and ProduXion Manager® from Aries Systems Corporation

Manuscript Click here to access/download;Manuscript;Manuscript.docx

Trends, Barriers and Enablers to Measles Immunisation Coverage in Saskatchewan, Canada: a Mixed Methods Study

Short title: Measles immunisation coverage in Saskatchewan, Canada

Marcus M Ilesanmi[1](ORCID: 0000-0003-2086-7923), Sylvia Abonyi[1,2] (ORCID: 0000-0003-2657-4144), Punam Pahwa[1,3](ORCID: 0000-0002-3342-0496), Volker Gerdts[4,5](ORCID: 0000-0001-8229-1611) Michael Scwandt[6,7](ORCID: 0000-0002-3164-8972), Cordell Neudorf[1,8] (ORCID: 0000-0003-4943-4785)

[1]Department of Community Health and Epidemiology, College of Medicine, University of Saskatchewan, Saskatoon, SK, Canada.

[2]Saskatchewan Population Health and Evaluation Research Unit (SPHERU), University of Saskatchewan, SK, Canada

[3]Canadian Centre for Health and Safety in Agriculture, University of Saskatchewan, Saskatoon, SK, Canada

[4]Vaccine and Infectious Disease Organization-International Vaccine Centre (VIDO-InterVac), University of Saskatchewan, Saskatoon, SK, Canada

[5]Department of Veterinary Microbiology, Western College of Veterinary Medicine, University of Saskatchewan, Saskatoon, SK, Canada

[6]University of British Columbia, School of Population and Public Health, Vancouver, BC, Canada

[7]Vancouver Coastal Health, Office of the Chief Medical Health Officer, Vancouver, BC, Canada

[8]Health Surveillance & Reporting, Saskatchewan Health Authority (SHA), Saskatoon, SK, Canada

***Corresponding Author:**

E-mail: marcus.ilesanmi@usask.ca (MMI)

Abstract

Many social, cultural, and systemic challenges affect the uptake of measles immunisation services. Prior studies have looked at the caregivers' perspectives, but little is known about the perspectives of the health care providers on the barriers of measles immunisation services in Canada. This study examined measles immunisation coverage trends across the regional health authorities in Saskatchewan and explored the barriers and enablers to measles immunisation coverage from providers' perspectives. The study adopted

an explanatory sequential mixed method. We utilized the entire population of 16,582 children under two years of age available in the Saskatchewan Immunisation Management System (SIMS) registry for 2002 and 2013 in aggregate format and interviewed 18 key informants in pre-determined two-stages in 2016 and 2017. The quantitative analysis was done with Joinpoint regression modelling, while the qualitative interview data was analyzed using hybrid inductive and deductive thematic approaches.

There was a 16.89%-point increase in measles immunisation coverage in the province from 56.32% to 73.21% between 2002 and 2013. There was also a persistently higher coverage among the affluent (66.95% - 82.37%) than the most deprived individuals (45.79% - 62.60%) in the study period. The annual rate of coverage change was marginally higher among the most deprived (16.81%; and average annual percentage change (AAPC) 2.0, 95% CI 1.7 – 2.2) than among the affluent group (15.42% and AAPC 3.0; 95% CI 2.0 – 4.0). While access-related issues, caregivers' fears, hesitancy, anti-vaccination challenges, and resource limitations were barriers to immunisation, improving community engagement, service delivery flexibility, targeted social responses and increasing media role were found useful to address the uptake of measles and other vaccine-preventable diseases immunisation challenges.

There is low coverage and inequity in measles immunisation uptake in Saskatchewan from social and institutional barriers. Even though there is evidence of disparity reduction among the different groups, the barriers to increasing measles immunisation coverage have implications for the health of the socio-economically deprived groups, the healthcare system and other vaccination programs. There is a need to improve policy framework for community engagement, targeted programs, and public health discourse.

Keywords

Measles; Immunisation coverage; Coverage disparity; Health providers perspective; Equity; Joinpoint regression.

Introduction

Among several infectious diseases, measles has received prominent attention internationally due to its high rate of infectivity [1-3]. Core to reducing the economic impact of diseases both on the health care financing and for children well-being is disease prevention achieved through immunisation, which has proved to be useful in the control and elimination of life-threatening infectious diseases.[4, 5] Measles immunisation averts between 2 and 3 million deaths globally each year [5, 6]. Between 2000 and 2018, 23.2 million deaths were prevented [7] resulting in approximately 73% drop in measles cases within that period. With all the gains, however, in 2019, there were 869 770 measles cases worldwide, being the highest number since 1996 and with about 207 500 (23.9%) lost lives in the same year.[8] Despite all the efforts to improve measles immunisation coverage, the proportion of immunised population with measles antigen containing vaccine (MCV) remains under the 95 percent herd immunity threshold (HIT).[8] A high percentage of unimmunized individuals portends high susceptibility and infectivity for vulnerable groups with unplanned public health expenditure and negative outcomes [9]. Considering the established linkage between social determinants of health and health outcomes,[10, 11] public health institutions are aware of the effect of health inequalities and inequities; however, evidence is not rife on whether these efforts have had impact on equity gaps.

Measles is a notifiable disease to be reported by health care providers in Canada as recommended in the 1924 national guideline.[12] The National Advisory Committee on Immunisation (NACI) emphasized the importance of elimination of indigenous measles since 1980,[13, 14] an objective which was reinforced by the Canadian Paediatric Association.[15] The Saskatchewan routine measles immunisation schedule are two doses of measles containing vaccine with first dose recommended at one year (12 months) and a second dose starting from 18 months of age.[16] In 1994, Health Canada joined other Pan American Ministries of Health to set year 2000 measles elimination target in the Western Hemisphere[13, 17, 18] Measles elimination was however achieved in 1998 when Canada was declared free of endemic measles

infection.[13, 18] Canada adopts 2-dose measles antigen-containing vaccination (MCV) recommendation by the World Health Organization (WHO),[19] however, with differential administration of the 2-dose approach where MCV1 is uniformly offered at 12 months, but varying timing of MCV2 across the ten provinces and three territories. Despite a strong institutional and organizational medical arrangement, immunisation is not mandatory at the national level in Canada. The provinces of Ontario, New Brunswick and Manitoba have implemented legislation that requires proof of immunisation for school entry[20] but immunisation, however, remains largely discretionary in other provinces. In many jurisdictions around the world, mandatory immunisation has been attempted for varying reasons with varying results, however, evidence did not support its effectiveness [21]. With the variations in the legal, policy and practice approach to immunisation, measles infection remains a source of concern in preventive medicine practice and research communities. Immunisation coverage rates measure the numbers of individuals who have received the appropriate doses by a specific date or age and are a reliable indicator of the preventative measures to control the spread of disease. Canada operates a goal of 95% coverage of one dose of measles immunisation by 2 years and 2 doses by 7 years [22]. The recent re-emergence of measles in some pockets of the population in Canada casts a doubt on possible elimination in the near term [23-25]. Some studies have linked the re-emergence with a low level of herd immunity threshold (HIT) for the disease. [26] In particular, low population coverage with the recommended 2-dose regimen for measles vaccine by age of 2 years has been flagged as a causal factor [27]. In the province of Saskatchewan, MCV1 coverage was 75.3% at 2 years and 91% for MCV2 at 7 years age in 2014,[28] a range of 63.6% - 86.4% for MCV2 among regional health authorities in 2016,[29] and 79.9% average (69.1% - 93.2%) in 2018.[30]

Differences in immunisation coverage within and between health regions or geographical areas have been documented in the literature [31-33] with differences in health arising from social determinants of health between different groups making it challenging for some individuals and groups to integrate fully in the society, which affect such individual’s health-seeking behaviour. Several factors influence the rate of

immunisation like poor access, low education, limited family support and poverty, religious beliefs and colony formation[34, 35] among others. Multiple chains of transmission have been documented among religious communities that actively oppose or resist immunisation efforts.[13, 36-38] Evidence from the Saskatoon Health Region (SHR) suggests that incomplete immunisation leading to low coverages is primarily associated with low income, single parenthood, cultural status, and differences in beliefs,[39] where immunisation disparities exist between rural and urban areas and from neighbourhood to neighbourhood.

Internationally, research into equity gaps in childhood immunisations exists but very little is known in a Saskatchewan context. Much focus have been given to coverage issues with little attention into timeliness of immunisation which is a key indicator for program performance. [40] Immunisation milestone delays can impact subsequent vaccines with possible protective compromise from vaccine-preventable diseases[41] Understanding trends in measles immunisation coverage as well as drivers of higher coverage in small-area geographies in comparison to other geographical sub regions can offer insight into how to improve immunisation policy development and implementation practices in the province of Saskatchewan and in contextually similar Canadian and international jurisdictions. Previous studies looking at immunisation uptake in the province of Saskatchewan have examined the perspectives of the caregivers [31], but little is known on the perspectives of the health care providers as an important contributor to understanding coverage issues. Publicly available statistics data show an unequal distribution of measles immunisation coverage among health regions in the province of Saskatchewan and within different geographical socio-economic quintiles. Apart from the large regional health authorities such as Saskatoon and Regina Qu'Appelle Health Regions, which had carried out studies to document the coverage within their jurisdictions, there is scanty information on coverage rates at sub-regional and provincial levels, or within various quintiles of deprivation in the rest of the province. This study therefore examines measles immunisation coverage among health regions in the Canadian province of

Saskatchewan and explores the barriers and enablers to achieving herd immunity threshold in the province and its smaller geographies.

Materials and Methods

Study Setting

The province of Saskatchewan is one of the thirteen provinces and territories of Canada. It is landlocked between the provinces of Manitoba and Alberta on the east and west respectively, Northwest Territories in the north and the United States of America to the south. It has an area of 651,900 km^2 and a population of 1.174 million inhabitants in 2019. The province currently operates through one health authority administratively. Until the consolidation of all regional health authorities (RHAs) into one Saskatchewan Health Authority in 2017, the health administration was carried out through Athabasca health authority located in the north of the province and twelve other health regions immediate to the south of Athabasca, namely Cypress, Five Hills, Heartland, Keewatin Yatthe, Kelsey trail, Mamawetan Churchill River, Prairie North, Prince Albert Parkland, Regina Qu'Appelle, Saskatoon, Sun Country, and Sunrise.[42]

Data sour[illegible]

The study adopts mixed methods of inquiry using historical measles immunisation quantitative data of 12-14 months and 18-24 months age groups and interviews of key informant in the province Saskatchewan. We extracted first dose MCV1 and second dose MCV2 from Saskatchewan Immunisation Management System (SIMS) registry from 2002 – 2013). This study took place at a period when health system transformation was taking place (i.e., from Saskatchewan Immunization Management System Registry to the Panorama Gateway). The only available data at the time of study were up to 2013, hence the study used the total sample available in the province of Saskatchewan Immunization Management Registry. SIMS registry is an electronic secure storage of health records of public health services in Saskatchewan which includes immunisation, communicable diseases, and outbreak management accessible by authorized public health providers. We used annualized data from the two population

groups aged 12-14 months and 18-24 months. We define immunisation coverage as the percentage of children who had their MCV1 between 12-14 months among the children population aged 12-14 months and percentage of children who had their MCV2 between 18-24 months among the children population aged 18-24 months. The two groups were chosen to conform with the Saskatchewan immunisation schedules.[16] The qualitative portion utilized interview data obtained from eighteen purposively selected participants interviewed at two different times. The interviewees included frontline officers and individuals with policy roles in their respective regional health authorities such as public health nurses, immunisation administrators and medical health officers (MHOs). All the selected eighteen key informants participated in the first phase of interviews while seventeen participated in the second phase. The first stage of interviews which took place in the fourth quarter of 2016 collected the interviewees' initial general perspectives on factors that promote or hinder measles immunisation uptake in their health regions while the second stage, in the fourth quarter of 2017, was conducted to validate key themes and insights from the initial interviews and after quantitative results were presented to the participants. The second interviews were carried out in-person and audiotaped for in-depth analysis. Qualitative data triangulation [43] was ensured by interviewing two stakeholders each from each regional health authority. The inclusion of the qualitative strand was to provide context and possible explanations to the quantitative findings while embedding the study results with the experiences of the participants [44, 45]. Eighty-three percent (n=15) of the interview participants had at least eleven years' experience, either in their present positions or in Saskatchewan RHAs in various public and population health capacities; with MHOs an average of thirteen years' experience and Coordinators/Front-line Immunization Officers, an average of twenty years. The information asked from the participants pertain to their work in those periods when the quantitative data were collected; hence they were reflecting on their experience during the time data were collected, taking into account the past and future implications. Fig 1 displays the ten participating health regions key informants' selection criteria

[Insert Fig 1 here]

Fig 1: Participants included in the Qualitative Strand of the Study

Ethical Consideration

This study was approved by the research ethics board of the University of Saskatchewan, Canada (BIO-16-18), Regina Qu'Appelle Health Region Research Ethics Board (REB-16-91) and all the remaining 9 participating Regional Health Authorities of the province of Saskatchewan. The immunisation records data was extracted with researcher-generated syntax from the Saskatchewan Immunisation Database through Saskatchewan eHealth in a special format to ensure that the data remained anonymized and de-identified throughout the research process and collated through a provincial PHO leveraging upon the province data sharing agreement. All key informants had both written and verbal informed consents before interviews.

Statistical analysis

The immunisation data was analysed using Joinpoint regression models methods. Joinpoint trend analysis regression software is useful to test whether an apparent change in trend over a time period is statistically significant or not [46] Our analysis seeks to determine whether there is an increase or decrease over time in coverage rates and assesses the rate of change and the behavior of a response variable separately in characteristics of groups in the different periods of the explanatory variable [47, 48] In this study, we evaluated the time trends in measles immunisation coverage by geographical location and socio-economic deprivation quintile characteristics. We compared rates of changes for the measles immunisation coverage by using the Annual Percent Change (APC) and Average Annual Percent Change (AAPC) and the confidence intervals to determine an increase or decrease trend depending on whether the APC is positive or negative. When the lower confidence limit has a positive value, the rate of change over the time-period is depicted to be an increase and a decrease if the upper confidence limit is negative.[48]

A ratio statistic was calculated to determine how the two groups relate to each other over the time period under observation, using R = (μ1 + 1) / (μ2 + 1), where APCs of the two groups are represented by μ1 and μ2 respectively [48]. The data generates outcome variables in percent coverage rate, and to use such data for Joinpoint regression, the percentages were converted to proportions, p. The standard error (SE) of the proportion was calculated using the formula below:

Standard Error (SE) for large sample sizes = sqrt [p x (1 – p) / n]

(where p is the proportion, n the total population of the observed)

The percent coverage data calculated for all the birth years (2002 – 2013) from each health region were used and the response variable for the analysis of coverage was the natural logarithm of the percent coverage. The independent variable was the year from 2002 to 2013. The analysis fitted the simplest Joinpoint model that the data allowed with a statistically significant Joinpoint set at $p<0.05$. For graphical displays and visualizations, we used STATA^{TM} 16 [49] to convert the Joinpoint results where necessary.

To assess socio-economic disparities, we used the index of deprivation developed at the Institut National de Sante Publique du Quebec (INSPQ) which measures deprivation at the level of dissemination areas (DAs), the smallest areas for which Census data are available in Canada, comprising of approximately 400 to 700 residents living in the same small area geography [50]. The deprivation index looked at social and material dimensions with the proportion of single parents, the proportion of residents living alone, and marital status in the social components while the material deprivation measured educational attainment, average income, and employment status variables.[50] The DAs were divided into five quintiles 1 to 5 (Q1 to Q5) where each quintile represented 20% of population with Q1 being the most privileged and Q5, the least. We referred to these quintiles as Socio-economic deprivation quintiles (S-EDQ1 – S-EDQ5) in this paper.

The qualitative analysis employed hybrid inductive and deductive thematic analysis strategy described by Fereday & Muir-Cochrane [51]. The interview data were transcribed verbatim and coded using Nvivo

version 12.[52] The transcribed data was read for familiarisation. Recurring words, patterns, similarities, and relationships were identified and grouped into key themes and linked to generate possible nodes. Categories that lacked adequate support and reasonable links in the data were excluded or integrated into themes with adequate codes or reported as outliers if they carry sufficient insight to address the study objectives and were hence reported in the study results and conclusions.

Results

Trends in measles immunisation

To understand the trends in measles immunisation in the province of Saskatchewan, we assessed the two-year age group. In the study population demographics (Fig 2), there's a progressive increase of the study population from 2002 to 2009 reaching the highest figure of 15,189 in 2009 and decreased gradually to 14,106 by 2013 but without reaching the lowest figure of 13,273 of 2002 (Fig 2A). Geographical location differences existed in Saskatchewan with about two-thirds in the cities as compared with one-third in the rural areas. There was also a progressive increase in the proportion of people living in the urban areas contrary to a gradual decline of population in the rural locations. Among the health regions, the RHAs with the three largest Saskatchewan cities, Saskatoon RHAs, Regina Qu'Appelle and Prince Albert Parkland saw a progressive increase in population in the study period, with Saskatoon RHA having a higher level of increase than Regina Qu'Appelle and Prince Albert Parkland RHAs. The other health regions observed either a constant or a reduction in population between 2002 and 2013. The proportion of <2-year children and the socio-economic quintile they belong to range between 13.77% for S-EDQ4 in 2011 to 20.28% for S-EDQ2 in 2013 (Fig 2B). All the five (1-5) levels of socio-economic status is represented in the province.

[Insert Fig 2 here]

Fig 2: Study Population Characteristics

From MCV2 results for 2002, Sun Country RHA had the highest coverage of 67.22% while Prairie North RHA had the lowest at 49.15%. In 2013, Sun Country attained the highest coverage figure of 84.2% while Prince Albert Parkland had the lowest coverage figure at 60.58% as compared with other RHAs. It should be noted, however, that there were varying coverage rates for each of the RHAs during the years 2002 – 2013. It is observed that Sun Country RHA reached a higher proportion of the target group than any other RHAs in the study period (Fig 3)

[Insert Fig 3 here]

Fig 3: Saskatchewan RHAs Measles Coverage among on-time for MCV2 at 24 months (2002 - 2013)

There was a progressive increase in MCV2 coverage in the province between 2002 – 2013 from 56.32% to 73.21% (16.89%-point increase) (Figure 4) The three segments represented by the APC were all significantly different from zero at 1.68, 4.09 and 2.14 for the periods 2002-2008, 2008-2011, and 2011-2013 respectively with an AAPC of 2.4 (95% C.I. 2.0 – 2.9, $P < .005$).

[Insert Fig 4 here]

Fig 4: Saskatchewan 10 RHAs average coverage for on-time for MCV2 at 24months (2002 - 2013)

Assessing disparity in measles coverage by geographical location

There was increase in the coverage trends in Saskatchewan rural and urban locations with coverage rates being higher in the rural than in the urban locations. The coverage rate among on-time for

second dose at 2-year (MCV2) increased between 2002 and 2008 (APC: 0.8, 95%CI: 01-1.6), followed by a higher progressive increase from 2008 till 2013 (APC: 3.0, 95%CI: 2.1 – 3.8) for the rural locations. The urban location coverage rates displayed an increase from 2002 – 2008 at an APC of 2.3, a further increase of APC of 4.5 from 2008 – 2011 with the rate of change being significant with 95%CIs of 1.7 – 3.0 and 1.6 – 7.5 respectively while the increase from 2011 – 2013 and an APC of 2.0, was not significant (95%CI -0.4 – 4.6). A test for parallelism of the two coverage rate trends for the on-time for MCV2 in the urban and rural locations to check for any difference between the two trend lines indicated no parallelism (p value = 0.027) indicating a significant difference between the trend lines of the two geographical locations.

[Insert Fig 5 here]

Fig 5: Trends of MCV2 coverage rates in 10 Saskatchewan RHAs by geographical location (2002–2013)

Assessing disparity in measles coverage by socio-economic status

To compare the relationship between the socio-economic status and measles coverage rates in the province, we used the result of a Joinpoint regression that models the coverage trends in Fig 5. Although we have presented the five socio-economic deprivation quintiles to see the relationships, we compare S-E DQ1 and 5 Joinpoint fitted models in this research paper on the RHA pooled data for on-time for second dose at 2-year age (MCV2). There was increase in coverage rates from 2002-2013 (APC 2.0) in the S-EDQ1, an increase that was significantly different from zero (CI of 1.7 – 2.2). The S-EDQ5 coverage rates with a three-section Joinpoints displayed an increase from 2002 – 2008 with an APC of 2.8, a further increase to 5.8 from 2008 – 2011. In both of those APCs (2002-2008 and 2008-2011), the rate of change was significant with CIs of 1.9 – 3.7 and 1.6 – 10.1 respectively. The third Joinpoint for the S-EDQ5 showed a decrease from 2011 – 2013 with an APC of -0.2, which was not significant (CI -3.8 – 3.5). The

two coverage rate trends for the on-time for second dose at 2-year age group in the S-EDQ1 and 5 were tested for parallelism (whether there was any difference between the two trend lines), and Parallelism was rejected (P<.001 at 95% CI).

[Insert Fig 6 here]

Fig 6: Trend of MCV2 coverage rates in 10 Saskatchewan RHAs by S-EDQs (2002–2013): S-EDQ – Socio-economic deprivation quintiles ranges from 1(affluent quintile) to 5 (poorest quintile).

Summarizing, there was progressive increase in the coverage rates for both S-EDQ 1 and 5 and among both rural and urban populations for MCV2 in the province of Saskatchewan. The coverage rates were higher in the S-EDQ1 than S-EDQ5 and among the rural dwellers than the urban inhabitants among the on-time for MCV2 all through 2002 – 2013. Both S-EDQ and geographical location trend lines show convergence in coverage from 2002 – 2011 before a divergence between 2011 – 2013.

Barriers to measles immunisation uptake

The interview responses show that many health regions struggle to realize adequate measles immunisation coverage rates due to different barriers, including individual, region-specific, and cross-cutting institutional factors. These various barriers are described here.

Access related issues:

Socio-economic factors impact the ability to bring the child to the clinic for immunisation due to lack of access to vehicles. Relatedly, it is reported that many new immigrants lack understanding of what health services are available and how to navigate the health system generally, and immunisation services specifically. Caregivers who are in the active workforce also have competing demands for their time, making it somewhat difficult to leave work to attend the clinic during work hours especially for those who are engaged in more than one job. The interviewees also mentioned transience as a challenge to achieving higher coverage. Transient populations include the First Nations population who have a place

of residence in the health regions but also live on-reserve and thus fall under the federal jurisdiction for health service provision. In Canada, First Nations people are often registered in the provincial system within the health regions data management platform as well as in the federal jurisdiction and are part of the denominator for coverage calculation. The systems level challenge to follow up with the immunisation status of this population is a major barrier to improving on the measles immunisation coverage rate in any of the health regions where this population exists because data are not shared routinely between federal and provincial jurisdictions.

Fear and hesitancy of caregivers

Personal factors such as fear, anxiety, and general skepticism of caregivers regarding the subject of immunisation was recurrent in the interviews. One of the most mentioned is "*fears and hesitancies of needles"* but also fears related to whether the measles vaccine causes autism or autism-related disorders, the possibility of reactions to the vaccine, distrust of the system, and parental concerns. According to participants, some parents sometimes wondered '*how many is enough or too much'* among the crowded immunisation schedules that children have to take at every milestone in their early years, especially when measles vaccine is given in combination with other antigens in MMR and MMRV. Such hesitations can hamper the optimization of coverage rates where lack of education and engagement is missing at the grassroots level.

Anti-Vaccination Issues

Related to the parental fear factor mentioned above is the prevalence of anti-vaccination ideology among certain segments of the population. While fear and anxiety are dependent on individual characteristics and can be mitigated with public education and thorough engagement strategies, anti-vaccination ideologies take a philosophical objection to immunisation in general, and to multiple vaccines for infants specifically. According to some interviewees, anti-vaxxers to the measles immunisation in Saskatchewan are sometimes influenced by unsubstantiated news in social media. Some participants also mentioned the potential role of collective cultural beliefs where people in communal living arrangements or tightly

organized religious, cultural, or social groups can exhibit “group-think” when anti-vaccination beliefs are promoted within the group. Participants identified several places in the province where local social or religious groups hold and promote anti-vaccine ideas where more targeted approaches to gaining trust may be required.

Systemic and resource limitations

The fourth set of barriers are related to institutional issues such as funding, staffing and media access. Interviewees are concerned about the shrinking funding of the health system in the province, which limits the amount of funds available to carry out public education at the community level, develop online platforms and materials to reach clients, and invest in transportation resources to reach remote settlements. While systemic and resource limitations impact all health regions, they amplify existing challenges in small, often remote geographical locations that are far from large urban areas, particularly those serving predominantly Indigenous populations. There is also the issue of data access restriction between the federal and provincial health jurisdictions, and across provincial health regions. Currently, First Nations data in the federal system are not readily available on provincial immunisation registries [53] even though there is movement between the on-reserve and the off-reserve jurisdictions. As of the time of the study interviews, each region could only look at their own data, even though the Ministry of Health could look at the entire database for the province. Therefore, when people move from one region to another, there can be delays in updating records making it difficult to get an accurate picture of who needs immunisation in that region for timely intervention. The integration of all health regions into ‘one health authority’ at the provincial level may have addressed this later challenge, however, the lack of access to the First Nations data with the federal system remains an issue. Interviewees reported that such barrier leads to inaccuracy in immunisation coverage data, inability to identify specific demographics for targeted outreach, and conduct a thorough analysis of resources required to achieve herd immunity.

Enablers to measles immunisation uptake

Interview participants identified a variety of enablers to mitigate current challenges to measles immunisation uptake in the province. Some of the codes and themes that emerged are in direct response to the barriers mentioned above—some of which are already in place in some health regions; however, they also highlight additional factors that could move the health authorities toward optimal measles immunisation uptake. These enablers are summarized below.

Improved communication and community engagement

Almost all interviewees stress the importance of multiple channels of communication and engagement that include a reminder system, interprofessional communication and active community and stakeholder engagement. Reminder methods such as telephone, letters, emails, cards and the CANImmunize app (formerly immunizeCA app) can be helpful for clients to track and keep their immunisation schedule. For example, one interviewee suggests *"once the child was 13 months old and they hadn't received their 1-year immunisation, they'd get a little card in the mail that reminded them they needed to book an appointment.*" Many health regions also promote the use of the CANImmunize app, an interactive app supported by the Public Health Agency of Canada to provide information about vaccines and immunisation frequently asked questions. Regular communication among health workers was also seen as an essential enabler for bridging knowledge gaps and to develop coordinated responses to client needs in order to build confidence in health care providers and the overall health system. In order to develop adequate knowledge, which is a prerequisite to organizational competency, training and workforce development require collaborations with other health care workers outside of the RHA, including physicians and Nurse Practitioners. At the individual level, some interviewees reported that the "handouts and pamphlets" provided at maternity wards upon delivery of babies have been helpful to engage new caregivers on immunisation conversations and to address any questions they may have. At the community level, community awareness outreach helps to stimulate conversation about measles immunisation especially in communities where they were beginning to see low attendance and uptake of immunisation services generally: "*We have gone to a couple of health fairs or promotional days where we actually take*

immunisation displays with us and just talk about not just measles but other vaccines". Interviewees also mentioned the need to coordinate communication with school districts through school nurses especially when the children have missed the opportunity to take the measles vaccines at the appropriate periods. For example, "*letters are sent home to the caregiver for consent to administer the vaccines that were due to older children*" These school reminders are helping to improve coverage for non-school age kids that are overdue for immunisation as they serve as triggers for caregivers about other children in their homes that are behind on routine immunisation.

Flexibility in service delivery

Another enabler identified by many interviewees is the need for flexibility in service delivery to accommodate different socioeconomic groups. These include flexible timing for services and creation of more drop-in clinics and sites for Low Socioeconomic (LSE) groups, some of whom may not own phones and hence telephone reminder messages may not reach them. Information tracking and evaluation is also linked to the perception of flexibility in service delivery. Many interviewees suggest running through overdue lists regularly, creating regular status reports of coverage to identify where to direct resources for maximum results. The number of sites, coverage status reports and client appointments are regularly reviewed and if necessary revised to support LSE clients. Through interactions with clients, health care workers also assess client attitudes that point to vaccine hesitancy so that the challenge can be addressed pro-actively: "*We are analyzing which areas of the region have low coverage like we have done in the rural areas as well, as I mentioned before, the hesitancy group which are in …. So, these are some cases where we are trying to identify whether it has to do with the access or might be some hesitant group that we want to go after.*"

Targeted social responses

In addition to communication, engagement and flexibility, some interviewees mentioned the necessity to address clients' other needs, including home visitation, provision of transportation or bus passes, rewards in the form of gift cards, where and when possible. According to aggregate interview responses, three

health regions are already providing these as enablers to increase access, although only two of the three health regions explicitly linked the practice to improved measles immunisation. In addition, these specific interviewees also indicated that different incentives work for different groups of people and suggested the need for each health region to understand their client characteristics before introducing any incentive. For example, "*incentives work actually better in lower socio-economic neighborhoods" and with "our inner-city population." "We would buy books for kids, and we have gifts cards from Safeway or Co-op that we would give to the parents"*.

Increasing role of the Media

While this is less frequent, some interviewees mentioned that the media has an important role to play in enabling measles and other forms of immunisation uptake in the province of Saskatchewan. "*We used it [the media] sometime for influenza and we had several people line up to get flu vaccine, even though we weren't appropriately staffed to manage the turnout, but we were happy to have the people... the media really does help"*. This includes the use of radio and TV to educate the public to encourage parents to immunize their children, and reiterates the importance of not only getting immunised but the timeliness of the immunisation: "*I think it would be great for the media to continue to supplement social media messages, but media does provide excellent service especially the radio stations,"* Despite the identification of a clear role for traditional media such as radio and TV, only a few interviewees mentioned the important role the social media can play and it is in regard to the fact that "*we have not explored the use of social media to their full potentials"*.

Discussion

In this paper, we present the measles immunisation coverage in the province of Saskatchewan focusing on the trends, barriers, and enablers to identify where actions are required for better uptake and herd immunity. The study used both quantitative and qualitative data to explore trends, patterns, and issues in the province and by extension to similar health jurisdictions in Canada and internationally. Figure 7

summarizes the results with various issues and clusters representing key building blocks—individual, social and institutional—that should be considered towards optimizing immunisation in the province. Each of these issues has critical implications for equity and public health policy. The framework includes individual, social and institutional dimensions of existing barriers and enablers and suggests a holistic, interconnected approach that integrates issues such as timeliness of measles immunisation dosages, population growth, urban-rural dichotomy, and regional differences and disparities in coverage rates.

[Insert Fig 7 here]

Fig 7: Policy and research interventions for measles immunisation uptake

Data trends and regional disparities

The practice of immunisation trends monitoring is primarily reliant on coverage rates for its estimation and utilizes data which are routinely collected as a proportion of prescribed target populations [54-57]. It is information from such coverage rates that are needed for required vaccine-needs projections and immunisation program planning and implementation, including the evaluation of progress toward both national and international goals. The coverage goal for childhood vaccines developed in 2017 was 95% at 24 months for all publicly funded vaccines in all Canadian provinces and territories [58]. This goal is consistent with the WHO, and 2012 Global Vaccine Action Plans which provided a framework for more equitable access to vaccines currently in use for the full benefits derivable from them [59], while reflecting the Canadian context which has been adopted since 1996. The trend in measles immunisation coverage across the ten health regions increased steadily from 2002 – 2013. The increase in trend may be a response to all the various health region-specific innovations and program implementation practices during the study period. While this is a positive sign, the HIT has not yet been reached, and in order to

reach it, regular reporting on coverage rate at small area level with targeted approaches to under-immunized sub-populations will be required.

Contextual adaption of interventions (incentives, targeted response)

Contextual adaptations were mentioned by interview participants as an enabler of immunisation coverage. Particularly, regional health authorities where targeted approaches are introduced observed improvement in immunisation uptake by the initiation and maintenance of community program builders recruited from the community to reach out to the parents and clients in the inner-city core neighborhoods in Saskatoon RHA in 2007. These targeted approaches are valued by individuals and communities, not only for measles immunisation effort but also for opportunities to address clients' other needs such as the determinants of the clients' overall well-being. This finding suggests that interventions and implementation practices do not have to take the form of 'one size fits all'; rather, the introduction of culturally relevant and contextually appropriate approaches may be more effective than a standardized, universal approach to client engagement and outreach. This approach is similar to what was described as proportionate universalism by Carey et al, 2015 [60]. The above efforts are part of the strategies and interventions that increased uptake of measles immunisation especially by bridging the gap in the socio-economic divide with a resultant improvement in the coverage rates over the period under study. These innovations and practices were confirmed by the key informant qualitative findings of this study. During the targeted approach period starting from 2007, many regional health authorities in the province of Saskatchewan utilized reminder systems more effectively, increased the number of immunisation service delivery points and became more flexible in terms of clinic opening hours to allow access for caregivers who could not attend within the strict regular opening hours. The reminder call systems have been used in other similar situations with improved coverage results.[61, 62] All these efforts are towards tailoring immunisation service to the targeted beneficiaries[63]. These approaches were employed in different combinations by RHAs in the past, currently and into the future with modifications depending on contexts.

Cross-regional knowledge-sharing, engagement, and collaborations

Prior to the implementation of "one-health authority" in the province of Saskatchewan, each of the 13 health regions operated a more siloed approach in terms of operational framework and data management. Community engagement and outreaches were tailored to each region's context and managed by health professionals assigned to manage immunisation programs. While some of the gaps associated with this approach may have been bridged, local adaptation of innovations to improve coverage rates according to local need is desirable but learning from what has worked in similar situations is best in speeding up the "diffusion of innovation". Both quantitative and qualitative data show some regions have better coverage rates due to geographical advantages and innovative practices. For example, while Regina and Saskatoon RHAs saw a progressive increase in coverage rates throughout the period under study, Sun Country attained the highest coverage figure of 84.2% for the on-time for 2nd dose measles vaccine. Flexibility in service delivery, a needs-based approach that considered client socio-economic position, incentivization, and proactive engagement with clients and communities that help health care workers to assess client attitudes that point to vaccine hesitancy are some of the areas where cross-regional knowledge sharing will be valuable to practice. This will require continuous collaboration with other health professionals, local communities and stakeholders, and decision-makers in planning, design and implementing initiatives that support such practices.

Prevalent institutional and policy issues

The study also found that institutional factors such as funding, staffing and database integration remain a barrier for measles immunisation uptake. This was particular the case for health regions covering rural, often-remote locations, which have transportation access challenges. In some cases, confusion about whether immunisation records of individuals in Indigenous communities are up-to-date due to lack of coordination and linkage between the federal and provincial immunisation registries exist. Increased funding could help bridge staffing and resource issues, and more easily encourage health care providers and individuals with policy roles to explore options for better coverage. The registry coordination challenge, however, is not new and has been raised in other similar studies, especially with respect to the

lack of access to First Nations immunisation data to calculate provincial coverage rates [53, 64]. Moreover, due to the mobility of some First Nations people between on-reserve and off-reserve homes, tracking and communicating immunisation updates may be duplicated or sometimes omitted. It is therefore imperative that a national immunisation registry is created to facilitate greater transparency, coordination and consistency in tracking coverage rates in provinces and to develop a more accurate, timely and meaningful national outlook for measles immunisation coverage [53].

Addressing psychological and philosophical issues

Social and community beliefs can discourage individuals from accessing measles immunisation, and health care providers do not have the authority and resources to address this challenge since immunisations are recommended and not mandated public health policy in many jurisdictions [65]. The scale of hesitancy and resistance is more pronounced in some religious communities but are also amplified by access to social media where ‘pseudo-science’ circulate more easily [66]. Several factors, including conspiracy theories, general distrust, belief in alternatives, and concerns about safety have been identified as the drivers of the resurgence of vaccine hesitancy in recent past [66]. Hospital admission for measles can be as high as 47% among unimmunised individuals who were age-eligible for measles immunisation and up to 53% may develop acute respiratory failure [67]. Health care providers can only do so much as the legal environment allows, which suggests that persuasion and education remain the only tools to engage ‘anti-vaxxers’. Therefore, there is need for a more coordinated approach within the health community and across government social sectors to engage individuals and communities where objection to measles immunisation is still prevalent to increase the profile of credible sources of vaccine safety information. Interview responses have established the benefits of the traditional media (TV and Radio) as a major enabler, but gaps still exist on the use of social media. The need for staff and client social media literacy skills and education therefore becomes more imperative and urgent to enable the public to decipher credible sources of information on measles immunisation.

Conclusion and public health implications

This study found that despite individual, social and institutional barriers to measles immunisation in the province of Saskatchewan, there has been a significant increase in uptake in all regional health authorities over the study period (2002-2013). However, the uptake is occurring at different average annual percent change in each region but with overall aggregate increase at the provincial level. While access related issues, fear and hesitancy of caregivers, anti-vaccination ideologies, and myriads of systemic and resource limitations were identified as key barriers in the province, enablers such as improved communication and community engagement, flexibility in service delivery, targeted social responses, and increasing role of the media are helping to address these issues. This suggests that there are opportunities for improvement, especially in the aspects of knowledge exchange, public engagement, and contextual adaptations of innovative or proven practices in a way that is sensitive to individual regional experience with measles immunisation. In the province of Saskatchewan, achieving HIT which is the desirable outcome of measles immunisation has not yet been reached, and for this to happen, regular reporting on coverage rate at small area level with targeted approaches to under-immunized sub-populations will be required.

While this research furthers existing knowledge on measles herd immunity threshold and can ultimately inform policy directions on measles immunisation, the results should be considered in light of its limitations. Firstly, only 10 of the 13 RHAs participated in the study. While these 10 RHAs represent 96.5% of the study population, the specific context, and characteristics of the non-participating RHAs may have been missed. Secondly, the Saskatchewan Immunisation Management System (SIMS) data used for the quantitative strand of the study was between 2002 and 2013—which could be considered relatively dated, although this range represented the entire dataset that was available in that system before Saskatchewan transitioned to the Panorama Gateway in 2014. However, the 2016 and 2017 qualitative data provided by key informants, majority of who were long serving in the RHAs were valuable in providing a comprehensive picture of trends and challenges faced in their respective RHAs addressing both interventions in the retrospective periods dating to a decade and how the new information will be

used to make changes in the future. An apparent limitation of interview data analysis is information recall bias. It should also be noted that data were not available for ethnicity and immigration status. Future studies are needed to explore patterns of measles immunisation among other sub-populations which frequently exhibit health inequities such as immigrants and indigenous population. Overall, measles immunisation remains an important public health issue, therefore, studying the trends and patterns in measles immunisation uptake and the associated barriers and enablers in a typical Canadian jurisdiction such as Saskatchewan is an important issue for public health discourse, policy, and research internationally.

Abbreviations

AAPC - Average Annual Percent Change; APC – Annual Percentage Change; HIT – Herd Immunity Threshold; LSE – Low Socioeconomic; MHO – Medical Health Officer; NACI – National Advisory Committee on Immunisation; RHA – Regional Health Authority; SDoH – Social Determinants of Health; S-EDQ – Socio-economic Deprivation Quintile; SIMS – Saskatchewan Immunisation Management System

Acknowledgements

MI would like to thank the University of Saskatchewan, Saskatoon, Saskatchewan, Canada for his doctoral support through the College of Medicine Graduate Award (CoMGRAD), National Science and Engineering Research Council (Integrated Training Program in Infectious Disease, Food Safety and Public Policy (ITraP) and Government of Saskatchewan Innovation and Opportunities Scholarship (SIOS). The content is solely the responsibility of the authors and does not necessarily reflect the official views of the supporters.

References

1. Moss WJ, Griffin DE: **Global measles elimination**. *Nature Reviews Microbiology* 2006, **4**(12):900.
2. Organization WH: **WHO guidelines for epidemic preparedness and response to measles outbreaks**. 1999.
3. CDC: **Measles--United States, 2004**. *JAMA* 2005, **54**(48):153-154.
4. WHO: **Vaccines**. 2015:Available from http://www.who.int/topics/vaccines/en/ accessed Dec 12, 2020.
5. Bbaale E: **Factors influencing childhood immunization in Uganda**. *J Health Popul Nutr* 2013, **31**(1):118-129.
6. Rosen G, Imperato PJ: **A history of public health**: JHU Press; 2015.
7. **Measles - Key Facts** [https://www.who.int/news-room/fact-sheets/detail/measles]
8. Patel MK, Goodson JL, Alexander JP Jr, Kretsinger K, Sodha SV, Steulet C, Gacic-Dobo M, Rota PA, McFarland J, Menning L *et al*: **Progress Toward Regional Measles Elimination — Worldwide, 2000–2019**. *MMWR Morb Mortal Wkly Report* 2020, **69**:1700-1705.
9. Lo NC, Hotez PJ: **Public Health and Economic Consequences of Vaccine Hesitancy for Measles in the United States**. *JAMA Pediatr* 2017, **171**(9):887-892.
10. WHO: **World Health Organization Commission on the Social Determinants of Health: social injustice is killing people on a grand scale**. *Scand J Public Health* 2008, **36**(8):896-897.
11. World Health Organisation: **Global Action on the Social Determinats of Health to address Health Equity**. 2015(Available at https://www.who.int/social_determinants/implementation/WHA68_sdh-resolution65-8-May2015.pdf accessed January 10, 2019).
12. Public Health Agency of Canada: **Guidelines for the prevention and control of measles outbreaks in Canada**. *Canada Communicable Disease Report* 2013, **39**.
13. Katz SL, King K, Varughese P, De Serres G, Tipples G, Waters J: **Measles elimination in Canada**. *Journal of Infectious Diseases* 2004, **189**(Supplement 1):S236-S242.
14. Katz SL, De Quadros CA, Izurieta H, Venczel L, Carrasco P: **Measles eradication in the Americas: progress to date**. *Journal of infectious diseases* 2004, **189**(Supplement 1):S227-S235.
15. White FM: **Policy for measles elimination in Canada and program implications**. *Reviews of infectious diseases* 1983, **5**(3):577-582.
16. Saskatchewan Government: **Routine Immunization Schedule**. 2018(Available from http://publications.gov.sk.ca/documents/13/17498-Web%20Routine%20Imms%20Schedules%20Dec.%202018.pdf Accessed December 24, 2020).
17. De Quadros C, Hersh B, Nogueira A, Carrasco P, Da Silveira C: **Measles eradication: experience in the Americas**. *Bulletin of the World Health Organization* 1998, **76**(Suppl 2):47.
18. Orenstein WA, Samuel KL, Hinman AR: **Summary and conclusions: measles elimination meeting, 16–17 March 2000**. *Journal of Infectious Diseases* 2004, **189**(Supplement 1):S43-S47.
19. World Health Organization: **WHO position on measles vaccines**. *Vaccine* 2009, **27**(52):7219-7221.
20. PHAC: **Canadian National Report on Immunization, 1996**. 1997, **Volume: 23S4**(Available https://www.canada.ca/en/public-health/services/reports-publications/canada-communicable-disease-report-ccdr/monthly-issue/2006-32/supplement-canadian-national-report-on-immunization-2006/canadian-national-report-on-immunization-2006.html accessed 31 March 2019).
21. MacDonald NE, Dubé E, Grandt D: **Childhood immunization programs**. *CCDR* 2020, **46**(7/8).
22. Government of Canada: **Vaccination Coverage Goals and Vaccine Preventable Disease Reduction Targets by 2025**. 2019(Available at https://www.canada.ca/en/public-

health/services/immunization-vaccine-priorities/national-immunization-strategy/vaccination-coverage-goals-vaccine-preventable-diseases-reduction-targets-2025.html#1.1.2 accessed 17 April 2019).

23. PHAC: **Canada Communicable DIsease Report**. 2013, **39**(Available from http://www.phac-aspc.gc.ca/publicat/ccdr-rmtc/13vol39/acs-dcc-4/index-eng.php accessed April 3, 2017).

24. Saskatoon Health Region: **Health status reporting**. 2014(Available https://www.saskatoonhealthregion.ca/locations_services/Services/Health-Observatory/Documents/Reports-Publications/2014_SHR_HSRfull_series2.pdf accessed 10 Mar, 2017).

25. PHAC: **Canada Communicable Disease Report**. 2016, **42-7**(Available at http://www.phac-aspc.gc.ca/publicat/ccdr-rmtc/16vol42/dr-rm42-7/ar-01-eng.php accessed April 11, 2017).

26. Fine P, Eames K, Heymann DL: **"Herd immunity": a rough guide**. *Clinical Infectious Diseases* 2011, **52**(7):911-916.

27. Pegorie M, Shankar K, Welfare W, Wilson R, Khiroya C, Munslow G, Fiefield D, Bothra V, McCann R: **Measles outbreak in Greater Manchester, England, October 2012 to September 2013: Epidemiology and Control**. *Eurosurveillance* 2014, **19**(49):20982.

28. PHAC: **Public Health Agency of Canada. (Week 5, 2015). Measles**. 2015(Available at: http://www.phac-aspc.gc.ca/im/vpd-mev/measles-rougeole-eng.php Accessed on Feb 14, 2015).

29. Saskatchewan Min. of Health: **Vaccine Preventable Disease Monitoring Report Diphtheria, 2015 and 2016** 2017(Available at http://publications.gov.sk.ca/documents/13/108145-Measles%202015%20%202016%20Report%2020170207.pdf accessed April 27, 2019).

30. Saskatchewan Government: **Childhood Immunization Coverage Statistics**. 2018(Available at http://publications.gov.sk.ca/documents/13/108019-Saskatchewan-Quarterly-Childhood-Immunization-Report-Jun-2018.pdf Accessed February 18, 2021).

31. Cushon JA, Neudorf CO, Kershaw TM, Dunlop TG, Muhajarine N: **Coverage for the entire population: Tackling immunization rates and disparities in Saskatoon Health Region**. *Canadian Journal of Public Health/Revue Canadienne de Sante'e Publique* 2012:S37-S41.

32. Singh PK: **Trends in child immunization across geographical regions in India: focus on urban-rural and gender differentials**. *PloS one* 2013, **8**(9):e73102.

33. Barker LE, Chu SY, Li Q, Shaw KM, Santoli JM: **Disparities between white and African-American children in immunization coverage**. *Journal of the National Medical Association* 2006, **98**(2):130.

34. Johnson AG: **A Geospatial Analysis: Low Childhood Immunization Rates in Alberta**. Faculty of Graduate Studies; 2014.

35. Cassel J: **Public health in Canada**. *The History of Public Health and the Modern State* 1994, **26**:276.

36. Dallaire F, De Serres G, Tremblay F-W, Markowski F, Tipples G: **Long-lasting measles outbreak affecting several unrelated networks of unvaccinated persons**. *Journal of Infectious Diseases* 2009, **200**(10):1602-1605.

37. Woudenberg T, van Binnendijk RS, Sanders EA, Wallinga J, de Melker HE, Ruijs WL, Hahné SJ: **Large measles epidemic in the Netherlands, May 2013 to March 2014: changing epidemiology**. *Eurosurveillance* 2017, **22**(3).

38. Phadke VK, Bednarczyk RA, Salmon DA, Omer SB: **Association between vaccine refusal and vaccine-preventable diseases in the United States: a review of measles and pertussis**. *JAMA* 2016, **315**(11):1149-1158.

39. Sundquist S. DT, Wright J., Findlater R., Grauer K: **2010 Annual Immunization Report**. In. Saskatoon: Saskatoon Health Region; 2011: 55.

40. Hull BP, McIntyre PB: **Timeliness of childhood immunisation in Australia**. *Vaccine* 2006, **24**(20):4403-4408.
41. Kiely M, Boulianne N, Talbot D, Ouakki M, Guay M, Landry M, Sauvageau C, De Serres G: **Impact of vaccine delays at the 2, 4, 6 and 12 month visits on incomplete vaccination status by 24 months of age in Quebec, Canada**. *BMC public health* 2018, **18**(1):1364.
42. **Saskatchewan Health Regions, 2017** [https://www150.statcan.gc.ca/n1/pub/82-402-x/2017001/maps-cartes/rm-cr10-eng.htm]
43. Guion LA, Diehl DC, McDonald D: **Triangulation: Establishing the validity of qualitative studies**. *Edis* 2011, **2011**(8):3-3.
44. Creswell JW, Creswell JD: **Research design: Qualitative, quantitative, and mixed methods approaches**: Sage publications; 2017.
45. Creswell JW, Klassen AC, Plano Clark VL, Smith KC: **Best practices for mixed methods research in the health sciences**. *Bethesda (Maryland): National Institutes of Health* 2011, **2013**:541-545.
46. Hoffman S, Propp JM, McCarthy BJ: **Temporal trends in incidence of primary brain tumors in the United States, 1985-1999**. *Neuro-oncology* 2006, **8**(1):27-37.
47. Clegg LX, Hankey BF, Tiwari R, Feuer EJ, Edwards BK: **Estimating average annual per cent change in trend analysis**. *Statistics in Medicine* 2009, **28**(29):3670-3682.
48. Dehkordi ZF, Tazhibi M, Babazade SJJoe, promotion h: **Application of joinpoint regression in determining breast cancer incidence rate change points by age and tumor characteristics in women aged 30–69 (years) and in Isfahan city from 2001 to 2010**. 2014, **3**.
49. **Stata version 16.1** [https://www.stata.com/ order/]
50. Pampalon R, Hamel D, Gamache P, Raymond G: **A deprivation index for health planning in Canada**. *Chronic Dis Can* 2009, **29**(4):178-191.
51. Fereday J, Muir-Cochrane E: **Demonstrating rigor using thematic analysis: A hybrid approach of inductive and deductive coding and theme development**. *International journal of qualitative methods* 2006, **5**(1):80-92.
52. **NVIVO** [https://www.qsrinternational.com/nvivo-qualitative-data-analysis-software/home]
53. Wilson SE, Quach S, MacDonald SE, Naus M, Deeks SL, Crowcroft NS, Mahmud SM, Tran D, Kwong JC, Tu K: **Immunization information systems in Canada: Attributes, functionality, strengths and challenges. A Canadian Immunization Research Network study**. *Canadian Journal of Public Health* 2016, **107**(6):e575-e582.
54. Lemeshow S, Robinson D: **Surveys to measure programme coverage and impact: a review of the methodology used by the expanded programme on immunization**. *World Health Stat Q* 1985, **38**(1):65-75.
55. Lim SS, Stein DB, Charrow A, Murray CJ: **Tracking progress towards universal childhood immunisation and the impact of global initiatives: a systematic analysis of three-dose diphtheria, tetanus, and pertussis immunisation coverage**. *The Lancet* 2008, **372**(9655):2031-2046.
56. Burton A, Monasch R, Lautenbach B, Gacic-Dobo M, Neill M, Karimov R, Wolfson L, Jones G, Birmingham M: **WHO and UNICEF estimates of national infant immunization coverage: methods and processes**. *Bulletin of the World Health Organization* 2009, **87**:535-541.
57. World Health Organization.: **The world health report: health systems financing: the path to universal coverage.** 2010(Available at http://whqlibdoc.who.int/whr/2010/9789241564021_eng.pdf accessed Jan 19, 2020).
58. Public Health Agency of Canada: **Vaccine Coverage in canadian Children**. In. Ottawa, Canada; 2018.
59. World Health Organization: **Global Vaccine Action Plan 2011 - 2020**. In. Geneva; 2012.

60. Carey G, Crammond B, De Leeuw E: **Towards health equity: a framework for the application of proportionate universalism**. *International journal for equity in health* 2015, **14**(1):81.
61. Jacobson Vann JC, Szilagyi P: **Patient reminder and recall systems to improve immunization rates**. *The Cochrane Library* 2005.
62. Bocquier A, Ward J, Raude J, Peretti-Watel P, Verger P: **Socioeconomic differences in childhood vaccination in developed countries: a systematic review of quantitative studies**. *Expert review of vaccines* 2017, **16**(11):1107-1118.
63. Thomas S, Cashman P, Islam F, Baker L, Clark K, Leask J, Butler R, Durrheim DN: **Tailoring immunisation service delivery in a disadvantaged community in Australia; views of health providers and parents**. *Vaccine* 2018, **36**(19):2596-2603.
64. Zinszer K, Tamblyn R, Bates DW, Buckeridge DL: **A qualitative study of health information technology in the Canadian public health system**. *BMC Public Health* 2013, **13**(1):1-7.
65. Lee C, Robinson JL: **Systematic review of the effect of immunization mandates on uptake of routine childhood immunizations**. *Journal of Infection* 2016, **72**(6):659-666.
66. Burki T: **Vaccine misinformation and social media**. *The Lancet Digital Health* 2019, **1**(6):e258-e259.
67. Lancella L, Di Camillo C, Vittucci A, Boccuzzi E, Bozzola E, Villani A: **Measles lessons in an anti-vaccination era: public health is a social duty, not a political option**. *Italian journal of pediatrics* 2017, **43**(1):102.

Supporting information

Appendices

S1 Appendix. First phase Qualitative Strand Interview Guide (Pre immunisation data analysis)

S2 Appendix. Second phase Qualitative Strand Interview Guide (After Immunisation data analysis and presentation of initial findings)

S1 Fig. Saskatchewan Geographical Distribution by proportion of <2year children (2002 – 2013)

 Click here to access/download;Figure;Fig 1.tif

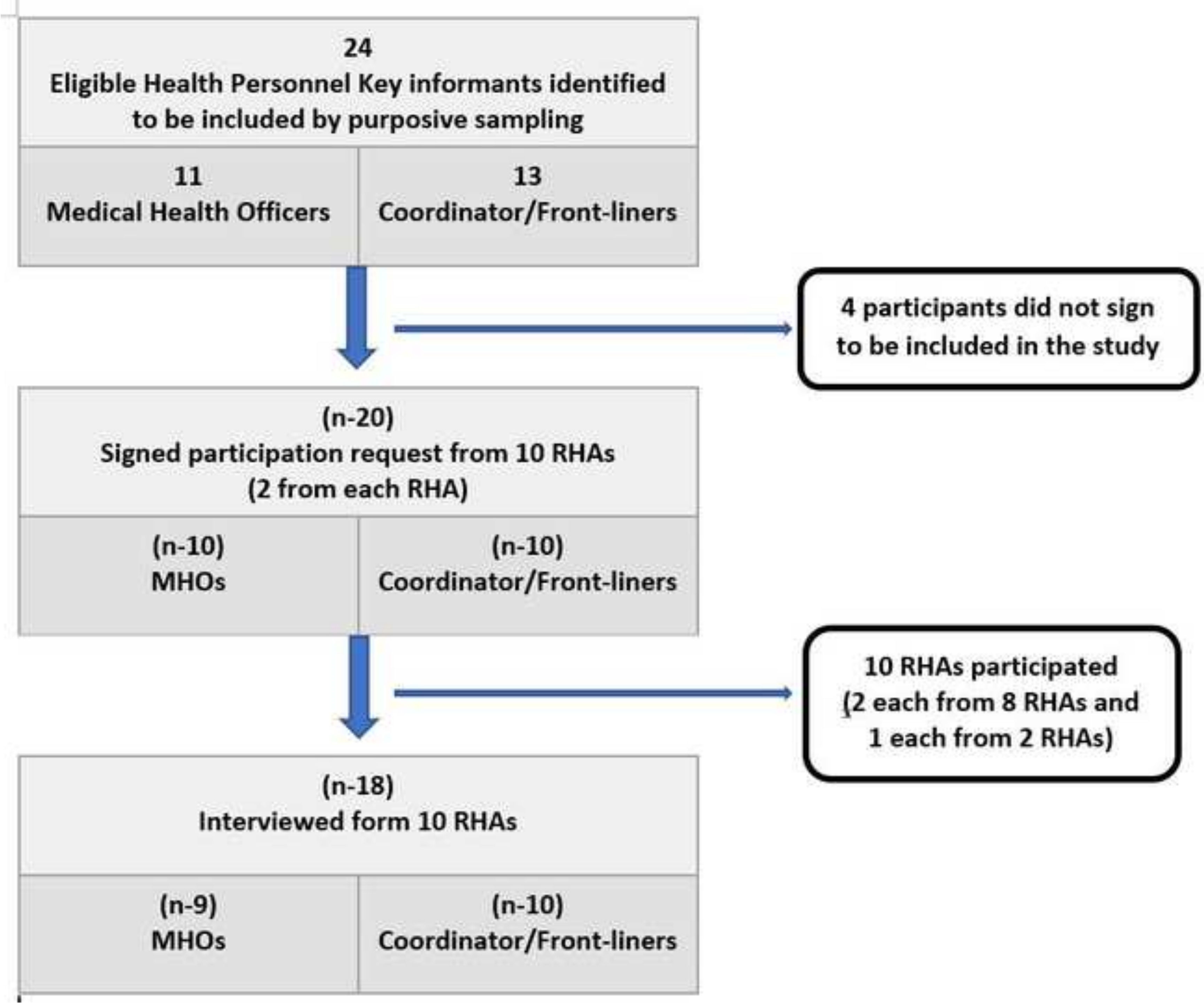

Figure 2

Click here to access/download;Figure;Fig 2.tif

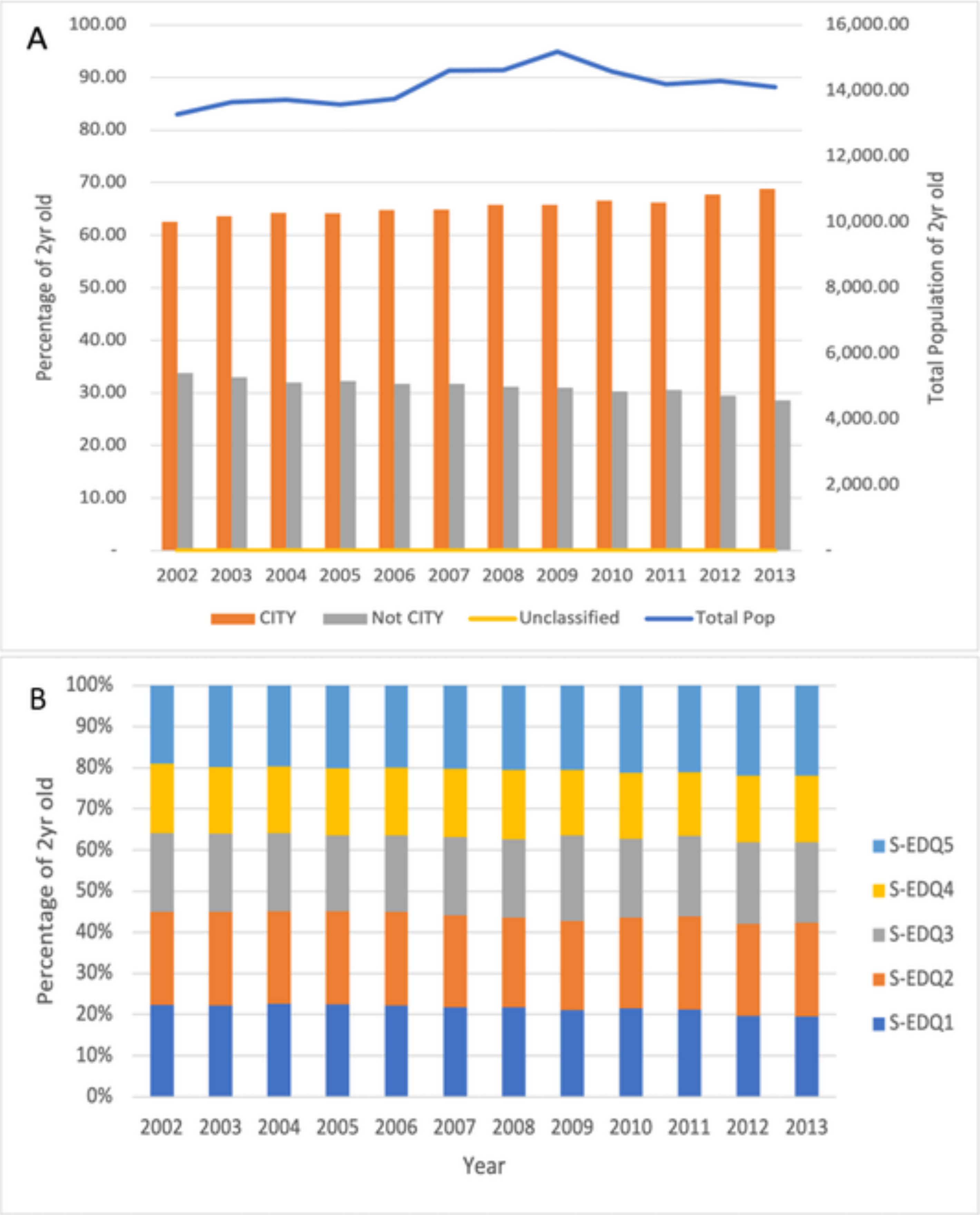

Figure 4

Click here to access/download;Figure;Fig 4.tif

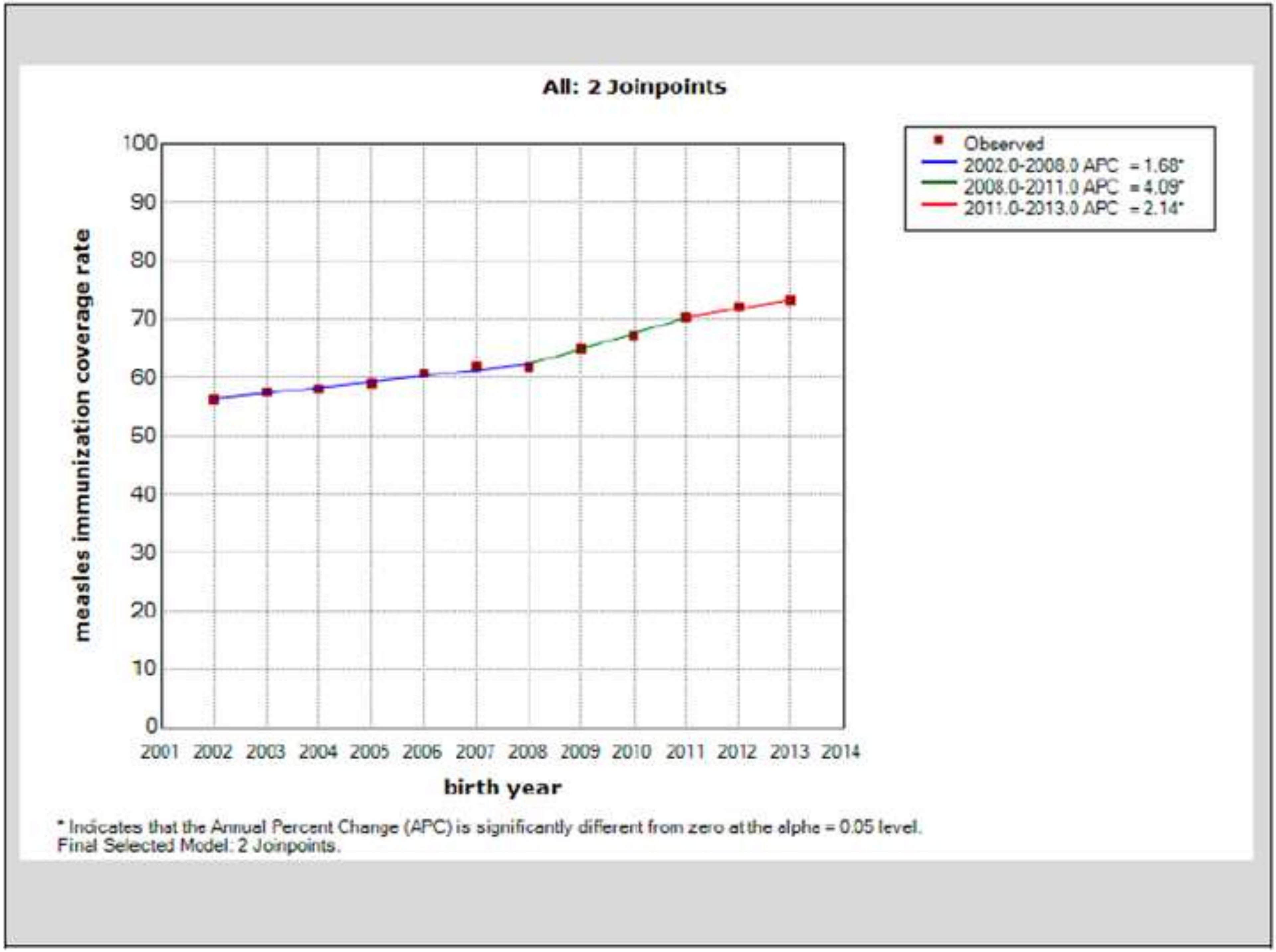

Figure 5

Click here to access/download;Figure;Fig 5.tif

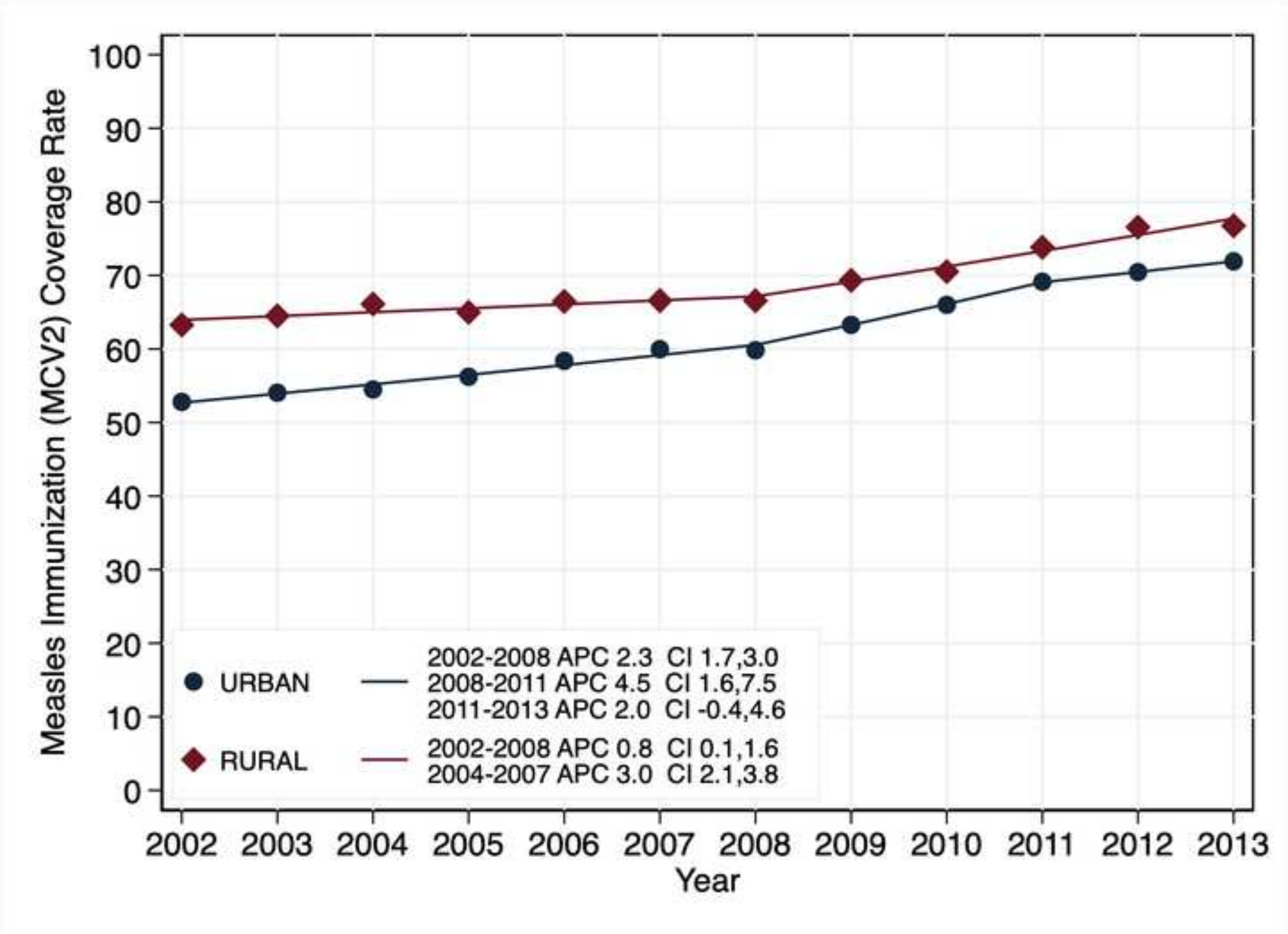

Figure 6

Click here to access/download;Figure;Fig 6.tif

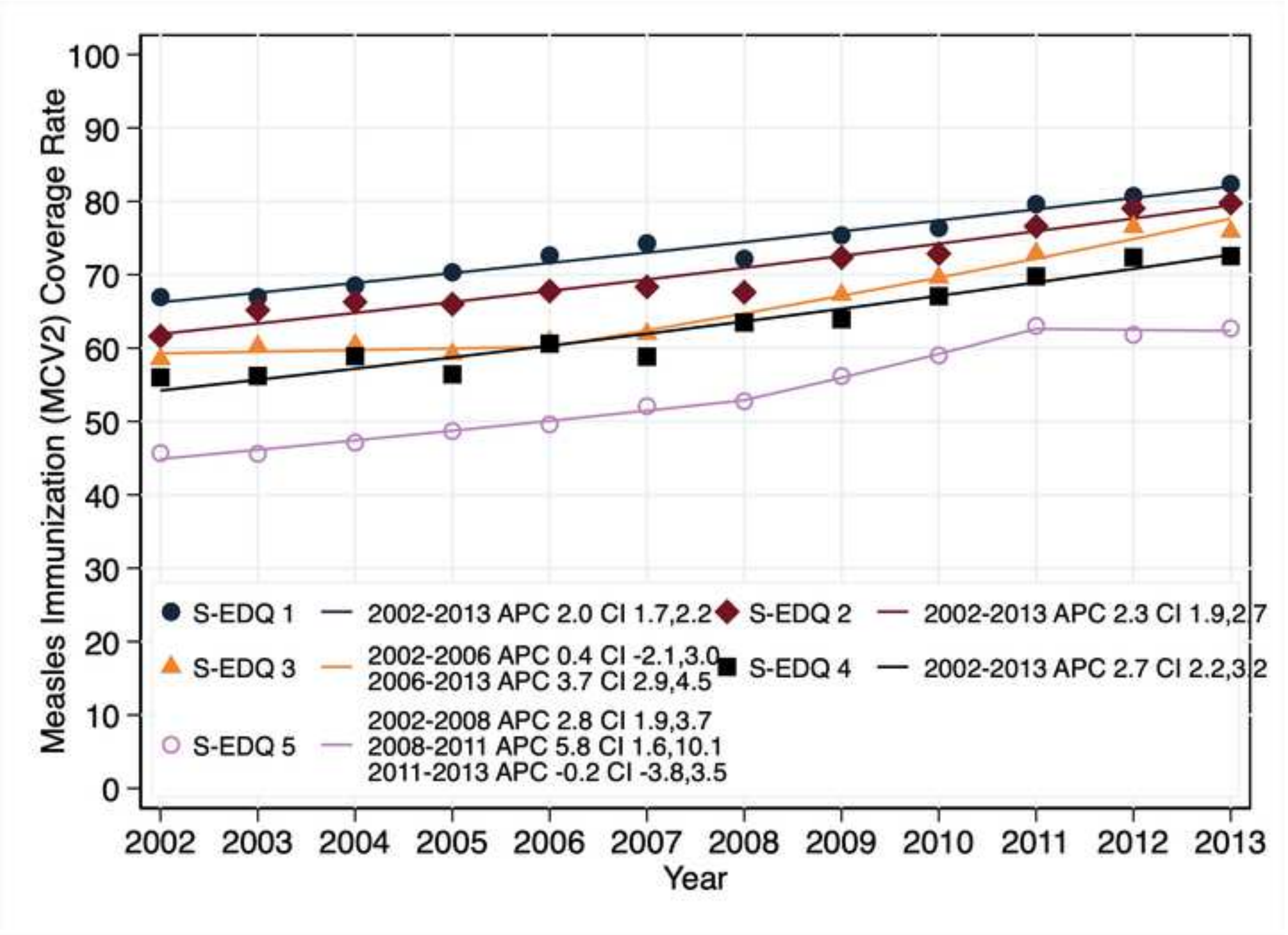

Figure 7

Click here to access/download;Figure;Fig 7.tif

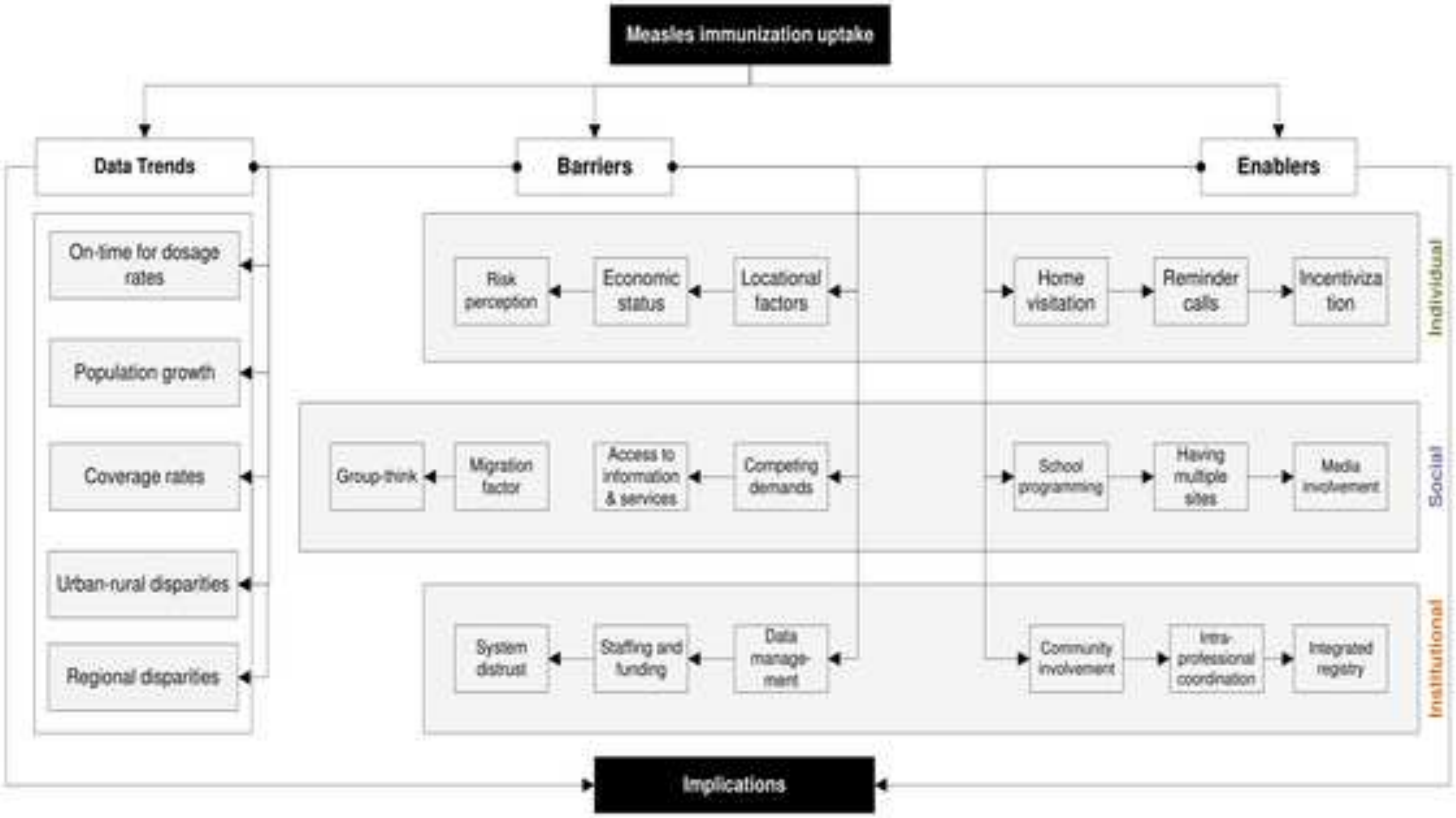

Click here to access/download
Supporting Information
Measles Immunisation Saskatchewan Supporting Information.docx

Trends, Barriers and Enablers to Measles Immunis~~z~~ation Coverage in Saskatchewan, Canada: a Mixed Methods Study

Short title: Measles immunis~~z~~ation coverage in Saskatchewan, Canada

Marcus M Ilesanmi[1](ORCID: 0000-0003-2086-7923), Sylvia Abonyi[1,2] (ORCID: 0000-0003-2657-4144), Punam Pahwa[1,3](ORCID: 0000-0002-3342-0496), Volker Gerdts[4,5](ORCID: 0000-0001-8229-1611) Michael Scwandt[6,7](ORCID: 0000-0002-3164-8972), Cordell Neudorf[1,8] (ORCID: 0000-0003-4943-4785)

[1]Department of Community Health and Epidemiology, College of Medicine, University of Saskatchewan, Saskatoon, SK, Canada.

[2]Saskatchewan Population Health and Evaluation Research Unit (SPHERU), University of Saskatchewan, SK, Canada

[3]Canadian Centre for Health and Safety in Agriculture, University of Saskatchewan, Saskatoon, SK, Canada

[4]Vaccine and Infectious Disease Organization-International Vaccine Centre (VIDO-InterVac), University of Saskatchewan, Saskatoon, SK, Canada

[5]Department of Veterinary Microbiology, Western College of Veterinary Medicine, University of Saskatchewan, Saskatoon, SK, Canada

[6]University of British Columbia, School of Population and Public Health, Vancouver, BC, Canada

[7]Vancouver Coastal Health, Office of the Chief Medical Health Officer, Vancouver, BC, Canada

[8]Health Surveillance & Reporting, Saskatchewan Health Authority (SHA), Saskatoon, SK, Canada

***Corresponding Author:**

E-mail: marcus.ilesanmi@usask.ca (MMI)

Abstract

Many social, cultural, and systemic challenges affect the uptake of measles immunisation services. Prior studies have looked at the caregivers' perspectives, but little is known about the perspectives of the health care providers on the barriers of measles immunis~~z~~ation services in Canada. This study ~~therefore~~ examined measles immunisation coverage trends across the regional health authorities in Saskatchewan and explored the barriers and enablers to measles immunis~~z~~ation coverage from providers' perspectives.

Formatted: Section start: Continuous, Header distance from edge: 0.49", Footer distance from edge: 0.49", Not Different first page header

Field Code Changed

Theeis study adopted an explanatory sequential mixed method. We utilized the entire population of 16,582 children under two years of age available in in the Saskatchewan Immunisszation Management System (SIMS) registry betweeforn between 2002 and 2013 in aggregate format and interviewed 18 key informants in pre-determined two-stages interviewsin 2016 and 20178. The quantitative analysis was done with Joinpoint regression modelling, while the qualitative interview data was analyzed using hybrid inductive and deductive thematic approaches.

There was a 16.89%-point increase in measles immunisation coverage in the province from 56.32% to 73.21% between 2002 and 2013. There was also a persistently higher coverage among the affluent (66.95% - 82.37%) than the most deprived individuals (45.79% - 62.60%) in the study period. The annual rate of coverage change was marginally higher among the most deprived (16.81%; and average annual percentage change (AAPC) 2.0, 95% CI 1.7 – 2.2) than among the affluent group (15.42% and AAPC 3.0; 95% CI 2.0 – 4.0). While access-related issues, caregivers' fears, hesitancy, anti-vaccination challenges, and resource limitations were barriers to immunisszation, improving community engagement, service delivery flexibility, targeted social responses and increasing media role were found useful to address the uptake of measles and other vaccine-preventable diseases immunisation challenges.

There is low coverage and inequity in measles immunisation uptake in Saskatchewan from social and institutional barriers. Even though there is evidence of disparity reduction among the different groups, the barriers to increasing measles immunisation coverage have implications for the health of the socio-economically deprived groups, the healthcare system and other vaccination programs. There is a need to improve policy framework for community engagement, targeted programs, and public health discourse.

Keywords

Measles; Immunisation coverage; Coverage disparity; Health providers perspective; Equity; Joinpoint regression.

Introduction

Among several infectious diseases, measles has received prominent attention internationally due to its high rate of infectivity [1-3]. Core to reducing the economic impact of diseases both on the health care financing and for children well-being is disease prevention achieved through immunisation, which has proved to be useful in the control and elimination of life-threatening infectious diseases.[4, 5] Measles immunisation averts between 2 and 3 million deaths globally each year [5, 6]. Between 2000 and 2018, 23.2 million deaths were prevented [7] resulting in approximately 73% drop in measles cases within that period. With all the gains, however, in 2019, there were 869 770 measles cases worldwide, being the highest number since 1996 and with about 207 500 (23.9%) lost lives in the same year.[8] Despite all the efforts to improve measles immunisation coverage, the proportion of immunised population with measles antigen containing vaccine (MCV) remains under the 95 percent herd immunity threshold (HIT).[8] A high percentage of unimmunized individuals portends high susceptibility and infectivity for vulnerable groups with unplanned public health expenditure and negative outcomes [9]. Considering the established linkage between social determinants of health and health outcomes,[10, 11] public health institutions are aware of the effect of health inequalities and inequities; however, evidence is not rife on whether these efforts have had impact on equity gaps.

Measles is ~~a reportable infection and~~ a notifiable disease to be reported by health care providers ~~since 1924~~ in Canada as ~~, and a~~ recommended in the 1924 national ~~objective~~guideline.[12] The National Advisory Committee on Immunis~~z~~ation (NACI) emphasized the importance of elimination of indigenous measles since~~in~~ 1980,[13, 14] an objective which was reinforced by the Canadian Paediatric Association.[15] The Saskatchewan routine measles immunisation schedule are two doses of measles containing vaccine with first dose recommended at one year (12 months) and a second dose starting from 18 ~~In~~months of age.[16] In 1994, Health Canada joined other Pan American Ministries of Health to set year 2000 measles elimination target in the Western Hemisphere[13, 17, 18] Measles elimination was

Formatted: Highlight

however achieved in 1998 when Canada was declared free of endemic measles infection.[13, 18] Canada adopts 2-dose measles antigen-containing vaccination (MCV) recommendation by the World Health Organization (WHO),[19] however, with differential administration of the 2-dose approach where MCV1 is uniformly offered at 12 months, but varying timing of MCV2 across the ten provinces and three territories. Despite a strong institutional and organizational medical arrangement, immunisation is not mandatory at the national level in Canada. The provinces of Ontario, New Brunswick and Manitoba have implemented legislation that requires proof of immunisation for school entry[20] but immunisation, however, remains largely discretionary in other provinces. In many jurisdictions around the world, mandatory immunisation has been attempted for varying reasons with varying results, however, evidence did not support its effectiveness [21]. With the variations in the legal, policy and practice approach to immunisation, measles infection remains a source of concern in preventive medicine practice and research communities. Immunisation coverage rates measure the numbers of individuals who have received the appropriate doses by a specific date or age and are a reliable indicator of the preventative measures to control the spread of disease. Canada operates a goal of 95% coverage of one dose of measles immunisation by 2 years and 2 doses by 7 years [22]. The recent re-emergence of measles in some pockets of the population in Canada casts a doubt on possible elimination in the near term [23-25]. Some studies have linked the re-emergence with a low level of herd immunity threshold (HIT) for the disease.[26] In particular, low population coverage with the recommended 2-dose regimen for measles vaccine by age of 2 years has been flagged as a causal factor [27]. In the province of Saskatchewan, MCV1 coverage was 75.3% at 2 years and 91% for MCV2 at 7 years age in 2014,[28] a range of 63.6% - 86.4% for MCV2 among regional health authorities in 2016,[29] and 79.9% average (69.1% - 93.2%) in 2018.[30]

Differences in immunisation coverage within and between health regions or geographical areas have been documented in the literature [31-33] with differences in health arising from social determinants of health between different groups making it challenging for some individuals and groups to integrate fully in the society, which affect such individual’s health-seeking behaviour. Several factors influence the rate of

immunisation like poor access, low education, limited family support and poverty, religious beliefs and colony formation[34, 35] among others. Multiple chains of transmission have been documented among religious communities that actively oppose or resist immunisation efforts.[13, 36-38] Evidence from the Saskatoon Health Region (SHR) suggests that incomplete immunisation leading to low coverages is primarily associated with low income, single parenthood, cultural status, and differences in beliefs,[39] where immunisation disparities exist between rural and urban areas and from neighbourhood to neighbourhood.

Internationally, research into equity gaps in childhood immunisations exists but very little is known in a Saskatchewan context. Much focus have been given to coverage issues with little attention into timeliness of immunisation which is a key indicator for program performance. [40] Immunisation milestone delays can impact subsequent vaccines with possible protective compromise from vaccine-preventable diseases[41] Also, much focus has been given to coverage issues, with little or no investigation into the on-time for age for measles immunisation. Timeliness of immunisation is a key indicator in program performance [40] and delays can impact subsequent vaccines and compromise protection from vaccine-preventable diseases[41]. Understanding trends in measles immunisation coverage as well as drivers of higher coverage in small-area geographies in comparison to other geographical sub regions can offer insight into how to improve immunisation policy development and implementation practices in the province of Saskatchewan and in contextually similar Canadian and international jurisdictions. Previous studies looking at immunisation uptake in the province of Saskatchewan have examined the perspectives of the caregivers [31], but little is known on the perspectives of the health care providers as an important contributor to understanding coverage issues. Publicly available statistics data show an unequal distribution of measles immunisation coverage among health regions in the province of Saskatchewan and within different geographical socio-economic quintiles. Apart from the large regional health authorities such as Saskatoon and Regina Qu'Appelle Health Regions, which had carried out studies to document the coverage within their jurisdictions, there is scanty information on coverage rates at sub-regional and

Formatted: Highlight

provincial levels, or within various quintiles of deprivation in the rest of the province. This study therefore examines measles immunisation coverage among health regions in the Canadian province of Saskatchewan and explores the barriers and enablers to achieving herd immunity threshold in the province and its smaller geographies.

Materials and Methods

Study Setting

The province of Saskatchewan is one of the thirteen provinces and territories of Canada. It is landlocked between the provinces of Manitoba and Alberta on the east and west respectively, Northwest Territories in the north and the United States of America to the south. It has an area of 651,900 km² and a population of 1.174 million inhabitants in 2019. The province currently operates through one health authority administratively. Until the consolidation of all regional health authorities (RHAs) into one Saskatchewan Health Authority in 2017, the health administration was carried out through Athabasca health authority located in the north of the province and twelve other health regions immediate to the south of Athabasca, namely Cypress, Five Hills, Heartland, Keewatin Yatthe, Kelsey trail, Mamawetan Churchill River, Prairie North, Prince Albert Parkland, Regina Qu'Appelle, Saskatoon, Sun Country, and Sunrise.[42]

Data source

The study adopts mixed methods of inquiry using historical measles immunisation quantitative data of 12-14 months and 18-24 months age groups and interviews of key informant ~~interviews~~ in the province Saskatchewan. We extracted first dose MCV1 and second dose MCV2 from ~~Panorama~~ Saskatchewan Immunisation Management System (SIMS) registry ~~for Saskatchewan~~ from 2002 – 2013 ~~(monthly and annual~~). This study took place at a period when health system transformation was taking place (i.e., from Saskatchewan Immunization Management System Registry to the Panorama Gateway). The only available data at the time of study were up to 2013, hence the study used the total sample available in the province of Saskatchewan Immunization Management Registry. ~~Panorama~~ SIMS registry is an electronic

Formatted: Font: (Default) Times New Roman, 11 pt, Font color: Auto

secure storage of health records of public health services in Saskatchewan which includes immunisation, communicable diseases, and outbreak management accessible by authorized public health providers. We used annualized data from the two population groups, aged 12-14 months and 18-24 months. We define immunisation coverage as the percentage of children who had their MCV1 between 12-14 months among the children population aged 12-14 months and percentage of children who had their MCV2 between 18-24 months among the children population aged 18-24 months. The two groups were chosen to conform with the Saskatchewan immunisation schedules.[16] The qualitative portion utilized interview data obtained from eighteen purposively selected participants interviewed at two different times. The iInterviewees included frontline officers and individuals with policy roles in their respective regional health authorities such as public health nurses, immunisation administrators and medical health officers (MHOs). All the selected eighteen18 key informants participated in the first phase of interviews and while seventeen17 participatednts in the second phase. The first stage of interviews which took place in the fourth quarter of 2016was to collectegatherd the interviewees' ir initial general perspectives on factors that promote or hinder MCV measles immunisation uptake in their health regions while the second stage, in the fourth quarter of 20178, was conducted to validate key themes and insights from the initial interviews and after quantitative results were presented to the participants. Both The sets ofsecond interviews were carried out in-person and audiotaped for in-depth analysis. Qualitative data triangulation[43] was ensured by interviewing two stakeholders each from each regional health authority. The inclusion of the qualitative strand was to provide context and possible explanations to the quantitative findings while embedding the study results with the experiences of the participants [44, 45]. Eighty-three percent (n=15) of the interview participants had at least eleven years' experience, either in their present positions or in Saskatchewan RHAs in various public and population health capacities; with MHOs an average of thirteen years' experience and Coordinators/Front-line Immunization Officers, an average of twenty years. The information asked from the participants pertain to their work in those periods when the quantitative data wereused was collected; hence they were reflecting on their experience during the time data wereas collected for, talking into account the past and future implicationsabout the past and up into

Formatted: Font: (Default) Times New Roman, English (United States)

Formatted: Font: (Default) Times New Roman, English (United States)

Formatted: Font: (Default) Times New Roman, English (United States)

Formatted: Font: (Default) Times New Roman, English (United States)

Formatted: Font: (Default) Times New Roman, English (United States)

Formatted: Font: (Default) Times New Roman, English (United States)

Formatted: Font: (Default) Times New Roman, English (United States)

Formatted: Font: (Default) Times New Roman, English (United States)

Formatted: Font: (Default) Times New Roman, English (United States)

Formatted: Font: (Default) Times New Roman, English (United States)

Formatted: Font: (Default) Times New Roman, 11 pt, Font color: Auto, English (United States)

Formatted: Font: (Default) Times New Roman, 11 pt, Font color: Auto, English (United States)

Formatted: Font: (Default) Times New Roman, 11 pt, Font color: Auto, English (United States)

Formatted: Font: (Default) Times New Roman, 11 pt, Font color: Auto, English (United States)

Formatted: Font: (Default) Times New Roman, 11 pt, Font color: Auto, English (United States)

Formatted: Font: (Default) Times New Roman, 11 pt, Font color: Auto, English (United States)

Formatted: Font: (Default) Times New Roman, 11 pt, Font color: Auto, English (United States)

Formatted: Font: (Default) Times New Roman, 11 pt, Font color: Auto, English (United States)

Formatted: Font: (Default) Times New Roman, 11 pt, Font color: Auto, English (United States)

Formatted: Font: (Default) Times New Roman, 11 pt, Font color: Auto, English (United States)

~~the future~~. Fig 1 displays the ten participating health regions key informants' selection ~~and the selection~~ criteria

Formatted: English (United States)

[Insert Fig 1 here]

Fig 1: Participants included in the Qualitative Strand of the Study

Ethical Consideration

This study was approved by the research ethics board of the University of Saskatchewan, Canada (BIO-16-18), Regina Qu'Appelle Health Region Research Ethics Board (REB-16-91) and all the remaining 9 participating Regional Health Authorities of the province of Saskatchewan. The immunisation records data was extracted with researcher-generated syntax from the Saskatchewan Immunisation Database through Saskatchewan eHealth in a special format to ensure that the data remained anonymized and de-identified throughout the research process and collated through a provincial PHO leveraging upon the province data sharing agreement. All key informants had both written and verbal informed consents before interviews.

Statistical analysis

The immunisation data was analysed using Joinpoint regression models methods. Joinpoint trend analysis regression software is useful to test whether an apparent change in trend over a time period is statistically significant or not [46] Our analysis seeks to determine whether there is an increase or decrease over time in coverage rates and assesses the rate of change and the behavior of a response variable separately in characteristics of groups in the different periods of the explanatory variable [47, 48] In this study, we evaluated the time trends in measles immunisation coverage by geographical location and socio-economic deprivation quintile characteristics. We compared rates of changes for the measles immunisation coverage by using the Annual Percent Change (APC) and Average Annual Percent Change (AAPC) and the confidence intervals to determine an increase or decrease trend depending on whether the APC is positive

or negative. When the lower confidence limit has a positive value, the rate of change over the time-period is depicted to be an increase and a decrease if the upper confidence limit is negative.[48]

A ratio statistic was calculated to determine how the two groups relate to each other over the time period under observation, using $R = (\mu 1 + 1) / (\mu 2 + 1)$, where APCs of the two groups are represented by $\mu 1$ and $\mu 2$ respectively [48]. The data generates outcome variables in percent coverage rate, and to use such data for Joinpoint regression, the percentages were converted to proportions, p. The standard error (SE) of the proportion was calculated using the formula below:

Standard Error (SE) for large sample sizes = sqrt [p x (1 – p) / n]

(where p is the proportion, n the total population of the observed)

The percent coverage data calculated for all the birth years (2002 – 2013) from each health region were used and the response variable for the analysis of coverage was the natural logarithm of the percent coverage. The independent variable was the year from 2002 to 2013. The analysis fitted the simplest Joinpoint model that the data allowed with a statistically significant Joinpoint set at $p<0.05$. For graphical displays and visualizations, we used STATA™ 16 [49] to convert the Joinpoint results where necessary.

Formatted: Superscript

To assess socio-economic disparities, we used the index of deprivation developed at the Institut National de Sante Publique du Quebec (INSPQ) which measures deprivation at the level of dissemination areas (DAs), the smallest areas for which Census data are available in Canada, comprising of approximately 400 to 700 residents living in the same small area geography [50]. The deprivation index looked at social and material dimensions with the proportion of single parents, the proportion of residents living alone, and marital status in the social components while the material deprivation measured educational attainment, average income, and employment status variables.[50] The DAs were divided into five quintiles 1 to 5 (Q1 to Q5) where each quintile represented 20% of population with Q1 being the most privileged and Q5, the least. We referred to these quintiles as Socio-economic deprivation quintiles (S-EDQ1 – S-EDQ5) in this paper.

The qualitative analysis employed hybrid inductive and deductive thematic analysis strategy described by Fereday & Muir-Cochrane [51]. The interview data were transcribed verbatim and coded using Nvivo version 12.[52] The transcribed data was read for familiarisation. Recurring words, patterns, similarities, and relationships were identified and grouped into key themes and linked to generate possible nodes. Categories that lacked adequate support and reasonable links in the data were excluded or integrated into themes with adequate codes or reported as outliers if they carry sufficient insight to address the study objectives and were hence reported in the study results and conclusions.

Results

Trends in measles immunisation

To understand the trends in measles immunisation in the province of Saskatchewan, we assessed the two-year age group. In the study population demographics (Fig 2), there's a progressive increase of the study population from 2002 to 2009 reaching the highest figure of 15,189 in 2009 and decreased gradually to 14,106 by 2013 but without reaching the lowest figure of 13,273 of 2002 (Fig 2A). Geographical location differences existed in Saskatchewan with about two-thirds in the cities as compared with one-third in the rural areas. There was also a progressive increase in the proportion of people living in the urban areas contrary to a gradual decline of population in the rural locations. Among the health regions, the RHAs with the three largest Saskatchewan cities, Saskatoon RHAs, Regina Qu'Appelle and Prince Albert Parkland saw a progressive increase in population in the study period, with Saskatoon RHA having a higher level of increase than Regina Qu'Appelle and Prince Albert Parkland RHAs. The other health regions observed either a constant or a reduction in population between 2002 and 2013. The proportion of <2-year children and the socio-economic quintile they belong to range between 13.77% for S-EDQ4 in 2011 to 20.28% for S-EDQ2 in 2013 (Fig 2B). All the five (1-5) levels of socio-economic status is represented in the province.

[Insert Fig 2 here]

Fig 2: Study Population Characteristics

From MCV2 results for 2002, In 2002, Sun Country health regionRHA had the highest coverage of 67.22% while Prairie North health regionRHA had the lowest at 49.15%. In 2013, Sun Country attained the highest coverage figure of 84.2% while Prince Albert Parkland had the lowest coverage figure at 60.58% as compared with other RHAs. It should be noted, however, that there were varying coverage rates for each of the health regionsRHAs during the years 2002 – 2013. It is observed that Sun Country RHA reached a higher proportion of the target group than any other RHAs in the study period (Fig 3)

[Insert Fig 3 here]

Fig 3: Saskatchewan RHAs Measles Coverage among on-time for MCV2 at 24 months (2002 - 2013)

There was a progressive increase in MCV2 coverage in the province between 2002 – 2013 from 56.32% to 73.21% (16.89%-point increase) (Figure 4) The three segments represented by the APC were all significantly different from zero at 1.68, 4.09 and 2.14 for the periods 2002-2008, 2008-2011, and 2011-2013 respectively with an AAPC of 2.4 (95% C.I. 2.0 – 2.9, P < .005).

[Insert Fig 4 here]

Fig 4: Saskatchewan 10 RHAs average coverage for on-time for MCV2 at 24months (2002 - 2013)

Assessing disparity in measles coverage by geographical location

There was increase in the coverage trends in Saskatchewan rural and urban locations with coverage rates being higher in the rural than in the urban locations. The coverage rate among on-time for second dose at 2-year (MCV2) increased between 2002 and 2008 (APC; 0.8, 95%CI: 01-1.6), followed by a higher progressive increase from 2008 till 2013 (APC; 3.0, 95%CI: 2.1 – 3.8) for the rural locations. The urban location coverage rates displayed an increase from 2002 – 2008 at an APC of 2.3, a further increase of APC of 4.5 from 2008 – 2011 with the rate of change being significant with 95%CIs of 1.7 – 3.0 and 1.6 – 7.5 respectively while the increase from 2011 – 2013 and an APC of 2.0, was not significant (95%CI -0.4 – 4.6). A test for parallelism of the two coverage rate trends for the on-time for MCV2 in the urban and rural locations to check for any difference between the two trend lines indicated no parallelism (p value = 0.027) indicating a significant difference between the trend lines of the two geographical locations.

[Insert Fig 5 here]

Fig 5: Trends of MCV2 coverage rates in 10 Saskatchewan RHAs by geographical location (2002–2013)

Assessing disparity in measles coverage by socio-economic status

To compare the relationship between the socio-economic status and measles coverage rates in the province, we used the result of a Joinpoint regression that models the coverage trends in Fig 5. Although we have presented the five socio-economic deprivation quintiles to see the relationships, we compare S-E DQ1 and 5 Joinpoint fitted models in this research paper on the RHA pooled data for ~~the on-time for first dose at 1-year and~~ on-time for second dose at 2-year age (MCV2)~~groups~~. ~~Among the on-time for first dose at 1-year age group, there was gentle but progressive increase in coverage rates between 2002 –~~

Formatted: Font: 11 pt

Formatted: Line spacing: Double

Formatted: Font: (Default) Times New Roman

Formatted: Font: (Default) Times New Roman

Formatted: Font: (Default) Times New Roman

Formatted: Font: (Default) Times New Roman

Formatted: Font: (Default) Times New Roman

Formatted: Font: (Default) Times New Roman

Formatted: Font: (Default) Times New Roman

Formatted: Font: (Default) Times New Roman

Formatted: Font: (Default) Times New Roman

Formatted: Caption, Indent: First line: 0", Line spacing: Double

~~2013 in the S-EDQ1 with an Annual Percentage Change (APC) 0.7 which was significantly different from zero at the alpha < 0.05 (CI 0.2 – 1.2). The S-EDQ5 coverage rates increased also through the period with an APC of 1.8 which was also significant (CI 1.3 – 2.3). The two trend lines in the Joinpoints models were tested for parallelism to see whether they were different, and parallelism was rejected (P<001), meaning the trends on coverage were different among both socio-economic deprivation quintiles.~~

~~Among the on-time for second dose at 2-year age group (MCV2), ~~T~~t~~here was increase in coverage rates from 2002-2013 (APC 2.0) in the S-EDQ1, an increase that was significantly different from zero (CI of 1.7 – 2.2). The S-EDQ5 coverage rates with a three-section Joinpoints displayed an increase from 2002 – 2008 with an APC of 2.8, a further increase to 5.8 from 2008 – 2011. In both of those APCs (2002-2008 and 2008-2011), the rate of change was significant with CIs of 1.9 – 3.7 and 1.6 – 10.1 respectively. The third Joinpoint for the S-EDQ5 showed a decrease from 2011 – 2013 with an APC of -0.2, which was not significant (CI -3.8 – 3.5). The two coverage rate trends for the on-time for second dose at 2-year age group in the S-EDQ1 and 5 were tested for parallelism (whether there was any difference between the two trend lines), and Parallelism was rejected (P<.001 at 95% CI).

[Insert Fig 6~~5~~ here]

Fig 6~~5~~: Trend of MCV2 coverage rates in 10 Saskatchewan RHAs by S-EDQs (2002–2013): S-EDQ – Socio-economic deprivation quintiles ranges from 1(affluent quintile) to 5 (poorest quintile). ~~(A) On-time for MCV1 (B) On-time for MCV2~~

Formatted: Space After: 0 pt, Line spacing: single

Summarizing, there was progressive increase in the coverage rates for both S-EDQ 1 and 5~~-~~ and among both rural and urban populations for MCV2 in the province of Saskatchewan.~~for both age groups studied (MCV1 and MCV2), the increase in coverage was significantly different from zero in both groups.~~ The coverage rates were higher in the S-EDQ1 than S-EDQ5 ~~for both on-time for MCV1 and~~and among the rural dwellers than the urban inhabitants among the on-time for MCV2 all through 2002 – 2013.~~ For on-time for MCV1,~~ Both ~~the~~ S-EDQ and geographical location trend lines show convergence in

coverage ~~for the period under study while the on-time for MCV2 showed a much larger degree of convergence~~ from 2002 – 2011 before a divergence between 2011 – 2013..

Barriers to measles immunisation uptake

The interview responses show that many health regions struggle to realize adequate measles immunisation coverage rates due to different barriers, including individual, region-specific, and cross-cutting institutional factors. These various barriers are described here.

Access related issues:

Socio-economic factors impact the ability to bring the child to the clinic for immunisation due to lack of access to vehicles. Relatedly, it is reported that many new immigrants lack understanding of what health services are available and how to navigate the health system generally, and immunisation services specifically. Caregivers who are in the active workforce also have competing demands for their time, making it somewhat difficult to leave work to attend the clinic during work hours especially for those who are engaged in more than one job. The interviewees also mentioned transience as a challenge to achieving higher coverage. Transient populations include the First Nations population who have a place of residence in the health regions but also live on-reserve and thus fall under the federal jurisdiction for health service provision. In Canada, First Nations people are often registered in the provincial system within the health regions data management platform as well as in the federal jurisdiction and are part of the denominator for coverage calculation. The systems level challenge to follow up with the immunisation status of this population is a major barrier to improving on the measles immunisation coverage rate in any of the health regions where this population exists because data are not shared routinely between federal and provincial jurisdictions.

Fear and hesitancy of caregivers

Personal factors such as fear, anxiety, and general skepticism of caregivers regarding the subject of immunisation was recurrent in the interviews. One of the most mentioned is "*fears and hesitancies of needles"* but also fears related to whether the measles vaccine causes autism or autism-related disorders,

the possibility of reactions to the vaccine, distrust of the system, and parental concerns. According to participants, some parents sometimes wondered '*how many is enough or too much'* among the crowded immunisation schedules that children have to take at every milestone in their early years, especially when measles vaccine is given in combination with other antigens in MMR and MMRV. Such hesitations can hamper the optimization of coverage rates where lack of education and engagement is missing at the grassroots level.

Anti-Vaccination Issues

Related to the parental fear factor mentioned above is the prevalence of anti-vaccination ideology among certain segments of the population. While fear and anxiety are dependent on individual characteristics and can be mitigated with public education and thorough engagement strategies, anti-vaccination ideologies take a philosophical objection to immunisation in general, and to multiple vaccines for infants specifically. According to some interviewees, anti-vaxxers to the measles immunisation in Saskatchewan are sometimes influenced by unsubstantiated news in social media. Some participants also mentioned the potential role of collective cultural beliefs where people in communal living arrangements or tightly organized religious, cultural, or social groups can exhibit "group-think" when anti-vaccination beliefs are promoted within the group. Participants identified several places in the province where local social or religious groups hold and promote anti-vaccine ideas where more targeted approaches to gaining trust may be required.

Systemic and resource limitations

The fourth set of barriers are related to institutional issues such as funding, staffing and media access. Interviewees are concerned about the shrinking funding of the health system in the province, which limits the amount of funds available to carry out public education at the community level, develop online platforms and materials to reach clients, and invest in transportation resources to reach remote settlements. While systemic and resource limitations impact all health regions, they amplify existing challenges in small, often remote geographical locations that are far from large urban areas, particularly

those serving predominantly Indigenous populations. There is also the issue of data access restriction between the federal and provincial health jurisdictions, and across provincial health regions. Currently, First Nations data in the federal system are not readily available on provincial immunisation registries [53] even though there is movement between the on-reserve and the off-reserve jurisdictions. As of the time of the study interviews, each region could only look at their own data, even though the Ministry of Health could look at the entire database for the province. Therefore, when people move from one region to another, there can be delays in updating records making it difficult to get an accurate picture of who needs immunisation in that region for timely intervention. The integration of all health regions into 'one health authority' at the provincial level may have addressed this later challenge, however, the lack of access to the First Nations data with the federal system remains an issue. Interviewees reported that such barrier leads to inaccuracy in immunisation coverage data, inability to identify specific demographics for targeted outreach, and conduct a thorough analysis of resources required to achieve herd immunity.

Enablers to measles immunisation uptake

Interview participants identified a variety of enablers to mitigate current challenges to measles immunisation uptake in the province. Some of the codes and themes that emerged are in direct response to the barriers mentioned above—some of which are already in place in some health regions; however, they also highlight additional factors that could move the health authorities toward optimal measles immunisation uptake. These enablers are summarized below.

Improved communication and community engagement

Almost all interviewees stress the importance of multiple channels of communication and engagement that include a reminder system, interprofessional communication and active community and stakeholder engagement. Reminder methods such as telephone, letters, emails, cards and the CANImmunize app (formerly immunizeCA app) can be helpful for clients to track and keep their immunisation schedule. For example, one interviewee suggests *"once the child was 13 months old and they hadn't received their 1-year immunisation, they'd get a little card in the mail that reminded them they needed to book an*

appointment.” Many health regions also promote the use of the CANImmunize app, an interactive app supported by the Public Health Agency of Canada to provide information about vaccines and immunisation frequently asked questions. Regular communication among health workers was also seen as an essential enabler for bridging knowledge gaps and to develop coordinated responses to client needs in order to build confidence in health care providers and the overall health system. In order to develop adequate knowledge, which is a prerequisite to organizational competency, training and workforce development require collaborations with other health care workers outside of the RHA, including physicians and Nurse Practitioners. At the individual level, some interviewees reported that the “handouts and pamphlets” provided at maternity wards upon delivery of babies have been helpful to engage new caregivers on immunisation conversations and to address any questions they may have. At the community level, community awareness outreach helps to stimulate conversation about measles immunisation especially in communities where they were beginning to see low attendance and uptake of immunisation services generally: “*We have gone to a couple of health fairs or promotional days where we actually take immunisation displays with us and just talk about not just measles but other vaccines”.* Interviewees also mentioned the need to coordinate communication with school districts through school nurses especially when the children have missed the opportunity to take the measles vaccines at the appropriate periods. For example, “*letters are sent home to the caregiver for consent to administer the vaccines that were due to older children*” These school reminders are helping to improve coverage for non-school age kids that are overdue for immunisation as they serve as triggers for caregivers about other children in their homes that are behind on routine immunisation.

Flexibility in service delivery

Another enabler identified by many interviewees is the need for flexibility in service delivery to accommodate different socioeconomic groups. These include flexible timing for services and creation of more drop-in clinics and sites for Low Socioeconomic (LSE) groups, some of whom may not own phones and hence telephone reminder messages may not reach them. Information tracking and evaluation is also

linked to the perception of flexibility in service delivery. Many interviewees suggest running through overdue lists regularly, creating regular status reports of coverage to identify where to direct resources for maximum results. The number of sites, coverage status reports and client appointments are regularly reviewed and if necessary revised to support LSE clients. Through interactions with clients, health care workers also assess client attitudes that point to vaccine hesitancy so that the challenge can be addressed pro-actively: "*We are analyzing which areas of the region have low coverage like we have done in the rural areas as well, as I mentioned before, the hesitancy group which are in …. So, these are some cases where we are trying to identify whether it has to do with the access or might be some hesitant group that we want to go after.*"

Targeted social responses

In addition to communication, engagement and flexibility, some interviewees mentioned the necessity to address clients' other needs, including home visitation, provision of transportation or bus passes, rewards in the form of gift cards, where and when possible. According to aggregate interview responses, three health regions are already providing these as enablers to increase access, although only two of the three health regions explicitly linked the practice to improved measles immunisation. In addition, these specific interviewees also indicated that different incentives work for different groups of people and suggested the need for each health region to understand their client characteristics before introducing any incentive. For example, "*incentives work actually better in lower socio-economic neighborhoods" and with "our inner-city population." "We would buy books for kids, and we have gifts cards from Safeway or Co-op that we would give to the parents"*.

Increasing role of the Media

While this is less frequent, some interviewees mentioned that the media has an important role to play in enabling measles and other forms of immunisation uptake in the province of Saskatchewan. "*We used it [the media] sometime for influenza and we had several people line up to get flu vaccine, even though we weren't appropriately staffed to manage the turnout, but we were happy to have the people... the media*

really does help”. This includes the use of radio and TV to educate the public to encourage parents to immunize their children, and reiterates the importance of not only getting immunised but the timeliness of the immunisation: “*I think it would be great for the media to continue to supplement social media messages, but media does provide excellent service especially the radio stations,”* Despite the identification of a clear role for traditional media such as radio and TV, only a few interviewees mentioned the important role the social media can play and it is in regard to the fact that “*we have not explored the use of social media to their full potentials”*.

Discussion

In this paper, we present the measles immunisation coverage in the province of Saskatchewan focusing on the trends, barriers, and enablers to identify where actions are required for better uptake and herd immunity. The study used both quantitative and qualitative data to explore trends, patterns, and issues in the province and by extension to similar health jurisdictions in Canada and internationally. Figure 7~~6~~ summarizes the results with various issues and clusters representing key building blocks—individual, social and institutional—that should be considered towards optimizing immunisation in the province. Each of these issues has critical implications for equity and public health policy. The framework includes individual, social and institutional dimensions of existing barriers and enablers and suggests a holistic, interconnected approach that integrates issues such as timeliness of measles immunisation dosages, population growth, urban-rural dichotomy, and regional differences and disparities in coverage rates.

[Insert Fig 7~~6~~ here]

Fig 7~~6~~: Policy and research interventions for measles immunisation uptake

Data trends and regional disparities

The practice of immunisation trends monitoring is primarily reliant on coverage rates for its estimation and utilizes data which are routinely collected as a proportion of prescribed target populations [54-57]. It is information from such coverage rates that are needed for required vaccine-needs projections and immunisation program planning and implementation, including the evaluation of progress toward both national and international goals. The coverage goal for childhood vaccines developed in 2017 was 95% at ~~at~~ 24 months for all publicly funded vaccines in all Canadian provinces and territories [58]. This goal is consistent with the WHO, and 2012 Global Vaccine Action Plans which provided a framework for more equitable access to vaccines currently in use for the full benefits derivable from them [59], while reflecting the Canadian context which has been adopted since 1996. The trend in measles immunisation coverage across the ten health regions increased steadily from 2002 – 2013. The increase in trend may be a response to all the various health region-specific innovations and program implementation practices during the study period. While this is a positive sign, the HIT has not yet been reached, and in order to reach it, regular reporting on coverage rate at small area level with targeted approaches to under-immunized sub-populations will be required.

Contextual adaption of interventions (incentives, targeted response)

Contextual adaptations were mentioned by interview participants as an enabler~~s~~ of immunisation coverage. Particularly, regional health authorities where targeted approaches are introduced observed improvement in immunisation uptake by the initiation and maintenance of community program builders recruited from the community to reach out to the parents and clients in the inner-city core neighborhoods in Saskatoon RHA in 2007. These targeted approaches are valued by individuals and communities, not only for measles immunisation effort but also for opportunities to address clients' other needs such as the determinants of the clients' overall well-being. This finding suggests that interventions and implementation practices do not have to take the form of 'one size fits all'; rather, the introduction of culturally relevant and contextually appropriate approaches may be more effective than a standardized,

universal approach to client engagement and outreach. This approach is similar to what was described as proportionate universalism by Carey et al, 2015 [60]. The above efforts are part of the strategies and interventions that increased uptake of measles immunisation especially by bridging the gap in the socio-economic divide with a resultant improvement in the coverage rates over the period under study. These innovations and practices were confirmed by the key informant qualitative findings of this study. During the targeted approach~~is~~ period starting from 2007, many regional health authorities in the province of Saskatchewan utilized reminder systems more effectively, increased the number of immunisation service delivery points and became more flexible in terms of clinic opening hours to allow access for caregivers who could not attend within the strict regular opening hours. The reminder call systems have been used in other similar situations with improved coverage results.[61, 62] All these efforts are towards tailoring immunisation service to the targeted beneficiaries[63]. These approaches were employed in different combinations by RHAs in the past, currently and into the future with modifications depending on contexts.~~The reminder call systems have been used in other similar situations with improved coverage results.~~[61, 62]

Cross-regional knowledge-sharing, engagement, and collaborations

Prior to the implementation of “one-health authority” in the province of Saskatchewan, each of the 13 health regions operated a more siloed approach in terms of operational framework and data management. Community engagement and outreaches were tailored to each region’s context and managed by health professionals assigned to manage immunisation programs. While some of the gaps associated with this approach may have been bridged, local adaptation of innovations to improve coverage rates according to local need is desirable but learning from what has worked in similar situations is best in speeding up the “diffusion of innovation”. Both quantitative and qualitative data show some regions have better coverage rates due to geographical advantages and innovative practices. For example, while Regina and Saskatoon RHAs saw a progressive increase in coverage rates throughout the period under study, Sun Country attained the highest coverage figure of 84.2% for the on-time for 2nd dose measles vaccine. Flexibility in

service delivery, a needs-based approach that considered client socio-economic position, incentivization, and proactive engagement with clients and communities that help health care workers to assess client attitudes that point to vaccine hesitancy are some of the areas where cross-regional knowledge sharing will be valuable to practice. This will require continuous collaboration with other health professionals, local communities and stakeholders, and decision-makers in planning, design and implementing initiatives that support such practices.

Prevalent institutional and policy issues

The study also found that institutional factors such as funding, staffing and database integration remain a barrier for measles immunisation uptake. This was particular the case for health regions covering rural, often-remote locations, which have transportation access challenges. In some cases, confusion about whether immunisation records of individuals in Indigenous communities are up-to-date due to lack of coordination and linkage between the federal and provincial immunisation registries exist. Increased funding could help bridge staffing and resource issues, and more easily encourage health care providers and individuals with policy roles to explore options for better coverage. The registry coordination challenge, however, is not new and has been raised in other similar studies, especially with respect to the lack of access to First Nations immunisation data to calculate provincial coverage rates [53, 64]. Moreover, due to the mobility of some First Nations people between on-reserve and off-reserve homes, tracking and communicating immunisation updates may be duplicated or sometimes omitted. It is therefore imperative that a national immunisation registry is created to facilitate greater transparency, coordination and consistency in tracking coverage rates in provinces and to develop a more accurate, timely and meaningful national outlook for measles immunisation coverage [53].

Addressing psychological and philosophical issues

Social and community beliefs can discourage individuals from accessing measles immunisation, and health care providers do not have the authority and resources to address this challenge since immunisations are recommended and not mandated public health policy in many jurisdictions [65]. The

scale of hesitancy and resistance is more pronounced in some religious communities but are also amplified by access to social media where ‘pseudo-science’ circulate more easily [66]. Several factors, including conspiracy theories, general distrust, belief in alternatives, and concerns about safety have been identified as the drivers of the resurgence of vaccine hesitancy in recent past [66]. Hospital admission for measles can be as high as 47% among unimmunised individuals who were age-eligible for measles immunisation and up to 53% may develop acute respiratory failure [67]. Health care providers can only do so much as the legal environment allows, which suggests that persuasion and education remain the only tools to engage ‘anti-vaxxers’. Therefore, there is need for a more coordinated approach within the health community and across government social sectors to engage individuals and communities where objection to measles immunisation is still prevalent to increase the profile of credible sources of vaccine safety information. Interview responses have established the benefits of the traditional media (TV and Radio) as a major enabler, but gaps still exist on the use of social media. The need for staff and client social media literacy skills and education therefore becomes more imperative and urgent to enable the public to decipher credible sources of information on measles immunisation.

Conclusion and public health implications

This study found that despite individual, social and institutional barriers to measles immunisation in the province of Saskatchewan, there has been a significant increase in uptake in all regional health authorities over the study period (2002-2013). However, the uptake is occurring at different average annual percent change in each region but with overall aggregate increase at the provincial level. While access related issues, fear and hesitancy of caregivers, anti-vaccination ideologies, and myriads of systemic and resource limitations were identified as key barriers in the province, enablers such as improved communication and community engagement, flexibility in service delivery, targeted social responses, and increasing role of the media are helping to address these issues. This suggests that there are opportunities for improvement, especially in the aspects of knowledge exchange, public engagement, and contextual adaptations of

innovative or proven practices in a way that is sensitive to individual regional experience with measles immunisation. In the province of Saskatchewan, achieving HIT which is the desirable outcome of measles immunisation has not yet been reached, and for this to happen, regular reporting on coverage rate at small area level with targeted approaches to under-immunized sub-populations will be required.

While this research furthers existing knowledge on measles herd immunity threshold and can ultimately inform policy directions on measles immunisation, the results should be considered in light of its limitations. Firstly, only 10 of the 13 RHAs participated in the study. While these 10 RHAs represent 96.5% of the study population, the specific context, and characteristics of the non-participating RHAs may have been missed. Secondly, the Saskatchewan Immunisation Management System (SIMS) data used for the quantitative strandaspect of the study was between 2002 and 2013—which could be considered relatively dated, although this range represented the entire dataset that was available in that system before Saskatchewan transitioned to the Panorama Gateway in 2014. However, the 2016 and 20178 qualitative data provided by key informants, majority of who were long serving in the RHAs which was collected in 2019, wereas valuable in providing a more comprehensivelete picture of trends and challenges facedissues in their respective RHAs addressing both interventions in the retrospective periods dating to a decade and how the new information will be used to make changes in the future. An apparent limitation of interview data analysis is information recall bias. It should also be noted that data were not available for ethnicity and immigration status. Future studies are needed to explore patterns of measles immunisation among other sub-populations which frequently exhibit health inequities such as immigrants and indigenous population. Overall, measles immunisation remains an important public health issue, therefore, studying the trends and patterns in measles immunisation uptake and the associated barriers and enablers in a typical Canadian jurisdiction such as Saskatchewan is an important issue for public health discourse, policy, and research internationally.

Abbreviations

AAPC - Average Annual Percent Change; APC – Annual Percentage Change; HIT – Herd Immunity Threshold; LSE – Low Socioeconomic; MHO – Medical Health Officer; NACI – National Advisory Committee on Immunisation; RHA – Regional Health Authority; SDoH – Social Determinants of Health; S-EDQ – Socio-economic Deprivation Quintile; SIMS – Saskatchewan Immunisation Management System

Acknowledgements

MI would like to thank the University of Saskatchewan, Saskatoon, Saskatchewan, Canada for his doctoral support through the College of Medicine Graduate Award (CoMGRAD), National Science and Engineering Research Council (Integrated Training Program in Infectious Disease, Food Safety and Public Policy (ITraP) and Government of Saskatchewan Innovation and Opportunities Scholarship (SIOS). The content is solely the responsibility of the authors and does not necessarily reflect the official views of the supporters.

References

1. Moss WJ, Griffin DE: **Global measles elimination**. *Nature Reviews Microbiology* 2006, **4**(12):900.
2. Organization WH: **WHO guidelines for epidemic preparedness and response to measles outbreaks**. 1999.
3. CDC: **Measles--United States, 2004**. *JAMA* 2005, **54**(48):153-154.
4. WHO: **Vaccines**. 2015:Available from http://www.who.int/topics/vaccines/en/ accessed Dec 12, 2020.
5. Bbaale E: **Factors influencing childhood immunization in Uganda**. *J Health Popul Nutr* 2013, **31**(1):118-129.
6. Rosen G, Imperato PJ: **A history of public health**: JHU Press; 2015.
7. **Measles - Key Facts** [https://www.who.int/news-room/fact-sheets/detail/measles]
8. Patel MK, Goodson JL, Alexander JP Jr, Kretsinger K, Sodha SV, Steulet C, Gacic-Dobo M, Rota PA, McFarland J, Menning L *et al*: **Progress Toward Regional Measles Elimination — Worldwide, 2000–2019**. *MMWR Morb Mortal Wkly Report* 2020, **69**:1700-1705.
9. Lo NC, Hotez PJ: **Public Health and Economic Consequences of Vaccine Hesitancy for Measles in the United States**. *JAMA Pediatr* 2017, **171**(9):887-892.
10. WHO: **World Health Organization Commission on the Social Determinants of Health: social injustice is killing people on a grand scale**. *Scand J Public Health* 2008, **36**(8):896-897.
11. World Health Organisation: **Global Action on the Social Determinats of Health to address Health Equity**. 2015(Available at https://www.who.int/social_determinants/implementation/WHA68_sdh-resolution65-8-May2015.pdf accessed January 10, 2019).
12. Public Health Agency of Canada: **Guidelines for the prevention and control of measles outbreaks in Canada**. *Canada Communicable Disease Report* 2013, **39**.
13. Katz SL, King K, Varughese P, De Serres G, Tipples G, Waters J: **Measles elimination in Canada**. *Journal of Infectious Diseases* 2004, **189**(Supplement 1):S236-S242.
14. Katz SL, De Quadros CA, Izurieta H, Venczel L, Carrasco P: **Measles eradication in the Americas: progress to date**. *Journal of infectious diseases* 2004, **189**(Supplement 1):S227-S235.
15. White FM: **Policy for measles elimination in Canada and program implications**. *Reviews of infectious diseases* 1983, **5**(3):577-582.
16. Saskatchewan Government: **Routine Immunization Schedule**. 2018(Available from http://publications.gov.sk.ca/documents/13/17498-Web%20Routine%20Imms%20Schedules%20Dec.%202018.pdf Accessed December 24, 2020).
17. De Quadros C, Hersh B, Nogueira A, Carrasco P, Da Silveira C: **Measles eradication: experience in the Americas**. *Bulletin of the World Health Organization* 1998, **76**(Suppl 2):47.
18. Orenstein WA, Samuel KL, Hinman AR: **Summary and conclusions: measles elimination meeting, 16–17 March 2000**. *Journal of Infectious Diseases* 2004, **189**(Supplement 1):S43-S47.
19. World Health Organization: **WHO position on measles vaccines**. *Vaccine* 2009, **27**(52):7219-7221.
20. PHAC: **Canadian National Report on Immunization, 1996**. 1997, **Volume: 23S4**(Available https://www.canada.ca/en/public-health/services/reports-publications/canada-communicable-disease-report-ccdr/monthly-issue/2006-32/supplement-canadian-national-report-on-immunization-2006/canadian-national-report-on-immunization-2006.html accessed 31 March 2019).
21. MacDonald NE, Dubé E, Grandt D: **Childhood immunization programs**. *CCDR* 2020, **46**(7/8).
22. Government of Canada: **Vaccination Coverage Goals and Vaccine Preventable Disease Reduction Targets by 2025**. 2019(Available at https://www.canada.ca/en/public-

health/services/immunization-vaccine-priorities/national-immunization-strategy/vaccination-coverage-goals-vaccine-preventable-diseases-reduction-targets-2025.html#1.1.2 accessed 17 April 2019).

23. PHAC: **Canada Communicable DIsease Report**. 2013, **39**(Available from http://www.phac-aspc.gc.ca/publicat/ccdr-rmtc/13vol39/acs-dcc-4/index-eng.php accessed April 3, 2017).

24. Saskatoon Health Region: **Health status reporting**. 2014(Available https://www.saskatoonhealthregion.ca/locations_services/Services/Health-Observatory/Documents/Reports-Publications/2014_SHR_HSRfull_series2.pdf accessed 10 Mar, 2017).

25. PHAC: **Canada Communicable Disease Report**. 2016, **42-7**(Available at http://www.phac-aspc.gc.ca/publicat/ccdr-rmtc/16vol42/dr-rm42-7/ar-01-eng.php accessed April 11, 2017).

26. Fine P, Eames K, Heymann DL: **"Herd immunity": a rough guide**. *Clinical Infectious Diseases* 2011, **52**(7):911-916.

27. Pegorie M, Shankar K, Welfare W, Wilson R, Khiroya C, Munslow G, Fiefield D, Bothra V, McCann R: **Measles outbreak in Greater Manchester, England, October 2012 to September 2013: Epidemiology and Control**. *Eurosurveillance* 2014, **19**(49):20982.

28. PHAC: **Public Health Agency of Canada. (Week 5, 2015). Measles**. 2015(Available at: http://www.phac-aspc.gc.ca/im/vpd-mev/measles-rougeole-eng.php Accessed on Feb 14, 2015).

29. Saskatchewan Min. of Health: **Vaccine Preventable Disease Monitoring Report Diphtheria, 2015 and 2016** 2017(Available at http://publications.gov.sk.ca/documents/13/108145-Measles%202015%20%202016%20Report%2020170207.pdf accessed April 27, 2019).

30. Saskatchewan Government: **Childhood Immunization Coverage Statistics**. 2018(Available at http://publications.gov.sk.ca/documents/13/108019-Saskatchewan-Quarterly-Childhood-Immunization-Report-Jun-2018.pdf Accessed February 18, 2021).

31. Cushon JA, Neudorf CO, Kershaw TM, Dunlop TG, Muhajarine N: **Coverage for the entire population: Tackling immunization rates and disparities in Saskatoon Health Region**. *Canadian Journal of Public Health/Revue Canadienne de Sante'e Publique* 2012:S37-S41.

32. Singh PK: **Trends in child immunization across geographical regions in India: focus on urban-rural and gender differentials**. *PloS one* 2013, **8**(9):e73102.

33. Barker LE, Chu SY, Li Q, Shaw KM, Santoli JM: **Disparities between white and African-American children in immunization coverage**. *Journal of the National Medical Association* 2006, **98**(2):130.

34. Johnson AG: **A Geospatial Analysis: Low Childhood Immunization Rates in Alberta**. Faculty of Graduate Studies; 2014.

35. Cassel J: **Public health in Canada**. *The History of Public Health and the Modern State* 1994, **26**:276.

36. Dallaire F, De Serres G, Tremblay F-W, Markowski F, Tipples G: **Long-lasting measles outbreak affecting several unrelated networks of unvaccinated persons**. *Journal of Infectious Diseases* 2009, **200**(10):1602-1605.

37. Woudenberg T, van Binnendijk RS, Sanders EA, Wallinga J, de Melker HE, Ruijs WL, Hahné SJ: **Large measles epidemic in the Netherlands, May 2013 to March 2014: changing epidemiology**. *Eurosurveillance* 2017, **22**(3).

38. Phadke VK, Bednarczyk RA, Salmon DA, Omer SB: **Association between vaccine refusal and vaccine-preventable diseases in the United States: a review of measles and pertussis**. *JAMA* 2016, **315**(11):1149-1158.

39. Sundquist S. DT, Wright J., Findlater R., Grauer K: **2010 Annual Immunization Report**. In. Saskatoon: Saskatoon Health Region; 2011: 55.

40. Hull BP, McIntyre PB: **Timeliness of childhood immunisation in Australia**. *Vaccine* 2006, **24**(20):4403-4408.
41. Kiely M, Boulianne N, Talbot D, Ouakki M, Guay M, Landry M, Sauvageau C, De Serres G: **Impact of vaccine delays at the 2, 4, 6 and 12 month visits on incomplete vaccination status by 24 months of age in Quebec, Canada**. *BMC public health* 2018, **18**(1):1364.
42. **Saskatchewan Health Regions, 2017** [https://www150.statcan.gc.ca/n1/pub/82-402-x/2017001/maps-cartes/rm-cr10-eng.htm]
43. Guion LA, Diehl DC, McDonald D: **Triangulation: Establishing the validity of qualitative studies**. *Edis* 2011, **2011**(8):3-3.
44. Creswell JW, Creswell JD: **Research design: Qualitative, quantitative, and mixed methods approaches**: Sage publications; 2017.
45. Creswell JW, Klassen AC, Plano Clark VL, Smith KC: **Best practices for mixed methods research in the health sciences**. *Bethesda (Maryland): National Institutes of Health* 2011, **2013**:541-545.
46. Hoffman S, Propp JM, McCarthy BJ: **Temporal trends in incidence of primary brain tumors in the United States, 1985-1999**. *Neuro-oncology* 2006, **8**(1):27-37.
47. Clegg LX, Hankey BF, Tiwari R, Feuer EJ, Edwards BK: **Estimating average annual per cent change in trend analysis**. *Statistics in Medicine* 2009, **28**(29):3670-3682.
48. Dehkordi ZF, Tazhibi M, Babazade SJJoe, promotion h: **Application of joinpoint regression in determining breast cancer incidence rate change points by age and tumor characteristics in women aged 30–69 (years) and in Isfahan city from 2001 to 2010**. 2014, **3**.
49. **Stata version 16.1** [https://www.stata.com/ order/]
50. Pampalon R, Hamel D, Gamache P, Raymond G: **A deprivation index for health planning in Canada**. *Chronic Dis Can* 2009, **29**(4):178-191.
51. Fereday J, Muir-Cochrane E: **Demonstrating rigor using thematic analysis: A hybrid approach of inductive and deductive coding and theme development**. *International journal of qualitative methods* 2006, **5**(1):80-92.
52. **NVIVO** [https://www.qsrinternational.com/nvivo-qualitative-data-analysis-software/home]
53. Wilson SE, Quach S, MacDonald SE, Naus M, Deeks SL, Crowcroft NS, Mahmud SM, Tran D, Kwong JC, Tu K: **Immunization information systems in Canada: Attributes, functionality, strengths and challenges. A Canadian Immunization Research Network study**. *Canadian Journal of Public Health* 2016, **107**(6):e575-e582.
54. Lemeshow S, Robinson D: **Surveys to measure programme coverage and impact: a review of the methodology used by the expanded programme on immunization**. *World Health Stat Q* 1985, **38**(1):65-75.
55. Lim SS, Stein DB, Charrow A, Murray CJ: **Tracking progress towards universal childhood immunisation and the impact of global initiatives: a systematic analysis of three-dose diphtheria, tetanus, and pertussis immunisation coverage**. *The Lancet* 2008, **372**(9655):2031-2046.
56. Burton A, Monasch R, Lautenbach B, Gacic-Dobo M, Neill M, Karimov R, Wolfson L, Jones G, Birmingham M: **WHO and UNICEF estimates of national infant immunization coverage: methods and processes**. *Bulletin of the World Health Organization* 2009, **87**:535-541.
57. World Health Organization.: **The world health report: health systems financing: the path to universal coverage.** 2010(Available at http://whqlibdoc.who.int/whr/2010/9789241564021_eng.pdf accessed Jan 19, 2020).
58. Public Health Agency of Canada: **Vaccine Coverage in canadian Children**. In. Ottawa, Canada; 2018.
59. World Health Organization: **Global Vaccine Action Plan 2011 - 2020**. In. Geneva; 2012.

60. Carey G, Crammond B, De Leeuw E: **Towards health equity: a framework for the application of proportionate universalism**. *International journal for equity in health* 2015, **14**(1):81.
61. Jacobson Vann JC, Szilagyi P: **Patient reminder and recall systems to improve immunization rates**. *The Cochrane Library* 2005.
62. Bocquier A, Ward J, Raude J, Peretti-Watel P, Verger P: **Socioeconomic differences in childhood vaccination in developed countries: a systematic review of quantitative studies**. *Expert review of vaccines* 2017, **16**(11):1107-1118.
63. Thomas S, Cashman P, Islam F, Baker L, Clark K, Leask J, Butler R, Durrheim DN: **Tailoring immunisation service delivery in a disadvantaged community in Australia; views of health providers and parents**. *Vaccine* 2018, **36**(19):2596-2603.
64. Zinszer K, Tamblyn R, Bates DW, Buckeridge DL: **A qualitative study of health information technology in the Canadian public health system**. *BMC Public Health* 2013, **13**(1):1-7.
65. Lee C, Robinson JL: **Systematic review of the effect of immunization mandates on uptake of routine childhood immunizations**. *Journal of Infection* 2016, **72**(6):659-666.
66. Burki T: **Vaccine misinformation and social media**. *The Lancet Digital Health* 2019, **1**(6):e258-e259.
67. Lancella L, Di Camillo C, Vittucci A, Boccuzzi E, Bozzola E, Villani A: **Measles lessons in an anti-vaccination era: public health is a social duty, not a political option**. *Italian journal of pediatrics* 2017, **43**(1):102.

Supporting information

Appendices

S1 Appendix. First phase Qualitative Strand Interview Guide (Pre immunisation data analysis)

S2 Appendix. Second phase Qualitative Strand Interview Guide (After Immunisation data analysis and presentation of initial findings)

S1 Fig. Saskatchewan Geographical Distribution by proportion of <2year children (2002 – 2013)

Authors’ response: Trends, Barriers and Enablers to Measles Immunization Coverage in Saskatchewan, Canada: a Mixed Methods Study

Reviewer #1: General comment
R1.1: The topic of the article proposed by the researchers is interesting, it is a contribution to the analysis of measles vaccination trends, from a mixed approach. The data is limited to the province of Saskatchewan but could be understood given the qualitative approach the authors add to the study.
Authors response: Thank you for your constructive comments
Changes made: None

R1.2: However, I am concerned about the lack of concurrency in the temporality of the quantitative and qualitative approaches. I can understand that the quantitative data for this study only includes up to the year 2013, while the qualitative analysis would correspond to a more current information collection (the authors do not report on this). If so, this temporal gap would mean that the social, historical and cultural context in which the analysis is carried out is not the same. Ideally, I would suggest to the authors to extend the trend analysis to a more current date, closer to the period in which the data from the qualitative approach were collected.
Authors response: We looked more into contextuality in this study. The study looked at data from 2002 to 2013 which were the available data in the Saskatchewan Immunization registry at the time of the study. We intentionally used data to make the key informants (mostly experienced managers) aware of the gap and or challenges in measles immunization coverage to have them pursue vigorously quality and equity initiatives. The first interview with the key informants took place in the third quarter of 2016 at which time the only available data on the SIMS registry were updated until 2013 and the interviews were with people who had been working in the system for many years and they reflected over that history and brought to fore what they have done in that period. The information pertaining to their work in those periods when the data were collected were asked. The information acquired were aimed at informing future interventions and practice. We conducted the first interview when they had not seen the findings from the quantitative analysis. The coverage rates that the RHAs had seen before our study was the overall coverage for their respective RHAs as made available by the Ministry of Health in a non-granular form like our study did. Our study looked at rural-urban disparity in coverages as well as different socio-economic groups. We then collated the generalized themes to see what were done in different RHAs and compared that with the outcome in terms of coverage. Subsequently, we shared the results of the quantitative analysis with the same group of interviewees to see if they have made improvements or not especially with new evidence and the new details on geographical and socio-economic analysis. With that level of analysis, we carried out knowledge translation with the informants. This led to the second stage interviews in 2018, when we interrogated the same set of people about the past and reflect on how they wanted to use the new information to make future programmatic changes. We think their responses reflected not just that point in time but their experience over the last decade and their future plans, therefore, there is no substantial gap that could have affected the results.
Changes made: Page 7, line 168 – 174: Eighty-three percent (n=15) of the interview participants had at least eleven years’ experience, either in their present positions or in Saskatchewan RHAs in various public and population health capacities; with MHOs, an average of thirteen years’ experience and Coordinators/Front-line Immunization Officers, an average of twenty years. The information asked from the participants pertained to their work in those periods when the quantitative data were collected; hence they were reflecting on their experience during the time data were collected, taking into account the past and future implications.
Also, page 23, lines 563 – 567: However, the 2016 and 2017 qualitative data provided by key informants, majority of who were long serving in the RHAs were valuable in providing a comprehensive picture of trends and challenges faced in their respective RHAs addressing both interventions in the retrospective periods dating to a decade and how the new information will be used to make changes in the future.

Summary
R1.3: Line 32. It is not clear in what time or periods of time the interviews with the key informants were taken.
Authors response: We have added the time of the interview
Changes made: Page 2, Lines 30 – 33: We utilized the entire population of 16,582 children under two years of age available in the Saskatchewan Immunization Management System (SIMS) registry for 2002 and 2013 in aggregate format and interviewed 18 key informants in pre-determined two-stages in 2016 and 2017.

Introduction
R1.4: Lines 75-78. Specify in the province of Saskatchewan, what is the regular schedule of measles vaccination in the first and second doses.
Authors' response: The routine schedule for first and second measles vaccination has been specified.
Change made: Page 3, lines 73 – 75: The Saskatchewan routine measles immunisation schedule are two doses of measles containing vaccine with first dose recommended at one year (12 months) and a second dose starting from 18 months of age.[1]

Materials and methods
R1.5: Line 145. Specify if the vaccination coverage was obtained: annually? For the entire province or for each of the regions?
Authors' response: As recommended, we have added "annualized"
Changes made: Page 6, line 151 – 152: We used annualized data from two population groups, aged 12-14 months and 18-24 months.

R1.6: Justify why a data analysis is performed only up to 2013, why more recent data is not included.
Authors' response: This study took place at a period when health system transformation was taking place hence, we used all the available data up to that point.
Change made: Page 6, lines 145 – 149: This study took place at a period when health system transformation was taking place (i.e., from Saskatchewan Immunization Registry to the Panorama Gateway). The only available data at the time of study were up to 2013, hence the study used the total sample available in the province of Saskatchewan Immunization Management Registry.

R1.7: Lines 153-156. Describe in what time period the key informant interviews were conducted.
Authors' response: The time period has been specified.
Change made: Page 7, lines 159 – 164: All the selected eighteen key informants participated in the first phase of interviews while seventeen participated in the second phase. The first stage of interviews which took place in the fourth quarter of 2016 collected the interviewees' initial general perspectives on factors that promote or hinder measles immunisation uptake in their health regions while the second stage, in the fourth quarter of 2017, was conducted to validate key themes and insights from the initial interviews and after quantitative results were presented to the participants

R1.8: Describe whether there was triangulation of qualitative information and how it was done.
Authors' response: To ensure consistency in information received from each of the health regions, we interviewed two key informants.
Change made: Page 7, line 165 – 166: Qualitative data triangulation was ensured by interviewing two stakeholders each from each regional health authority.

R1.9: How do the authors justify a quantitative analysis of data from 2010-2013, while the qualitative analysis corresponds to a current period of time? That is, although there is a mixed approach to the research, the authors have worked with a quantitative and qualitative approach at two different, non-concurrent moments.
Authors' response: See response above (R1.2)
Change made: Response R1.2 above

Statistical analysis
R1.10: An analysis of differences by socioeconomic quintiles is carried out in the results. It is necessary to describe in the Statistical analysis section where this information was obtained and how it was included in the analysis.
Authors' response: We have described how socioeconomic quintiles were estimated in the methods section
Changes made: Page 9, lines 212 – 221: To assess socio-economic disparities, we used the index of deprivation developed at the Institut National de Sante Publique du Quebec (INSPQ) which measures deprivation at the level of dissemination areas (DAs), the smallest areas for which Census data are available in Canada, comprising of approximately 400 to 700 residents living in the same small area geography[50]. The deprivation index looked at social and material dimensions with the proportion of single parents, the proportion of residents living alone, and marital status in the social components while the material deprivation measured educational attainment, average income, and employment status variables.[50] The DAs were divided into five quintiles 1 to 5 (Q1 to Q5) where each quintile represented 20% of population

with Q1 being the most privileged and Q5, the least. We referred to these quintiles as Socio-economic deprivation quintiles (S-EDQ1 – S-EDQ5) in this paper.

R1.11: I consider that, when evaluating the disparity in measles coverage by socioeconomic status, the authors could delve deeper into the results, there are some inequalities analyzes that could perhaps be applied in the present study, as well as exploring differences by urban, rural, between migrants, between indigenous and non-indigenous populations
Authors' response: Your suggestions are well noted. We have included information on urban and rural analysis in the results section as well as in the discussion. However, immunization data by ethnicity and immigration status were not systematically collected in the province of Saskatchewan. Also, the authors did not have access to individual but aggregated data for the study. We have hence noted this as part of study limitations.
Changes made: Pages 11 – 12, lines 268 – 278:
Assessing disparity in measles coverage by geographical location
There was increase in the coverage trends in Saskatchewan rural and urban locations with coverage rates being higher in the rural than in the urban locations. The coverage rate among on-time for second dose at 2-year (MCV2) increased between 2002 and 2008 (APC: 0.8, 95%CI:), followed by a higher progressive increase from 2008 till 2013 (APC: 3.0, 95%CI:) for the rural locations. . The urban location coverage rates displayed an increase from 2002 – 2008 at an APC of 2.3, a further increase of APC of 4.5 from 2008 – 2011 with the rate of change being significant with CIs of 1.7 – 3.0 and 1.6 – 7.5 respectively while the increase from 2011 – 2013 and an APC of 2.0, was not significant (CI -0.4 – 4.6). A test for parallelism of the two coverage rate trends for the on-time for MCV2 in the urban and rural locations to check for any difference between the two trend lines indicated no parallelism (p-value = 0.027) indicating a significant difference between the trend lines of the two geographical locations.
Page 13, lines 301 – 305: Summarizing, there was progressive increase in the coverage rates for both S-EDQ 1 and 5 and among both rural and urban populations for MCV2 in the province of Saskatchewan. The coverage rates were higher in the S-EDQ1 than S-EDQ5 and among the rural dwellers than the urban inhabitants among the on-time for MCV2 all through 2002 – 2013.
Page 24, line 568 – 570: It should be noted that data were not available for ethnicity and immigration status. Future studies are needed to explore patterns of measles immunization among other sub-populations which frequently exhibit inequities such as immigrants and indigenous population.

Results

R1.12: Lines 223-224. Are you referring to first or second dose coverage for measles, or full schedule coverage?
Authors' response: We have included that second dose for measles (MCV2) was used which is also the full schedule according to World Health Organisation (WHO).
Change made: Page 11, line 250 – 251: From MCV2 results for 2002, Sun Country RHA had the highest coverage of 67.22% while Prairie North RHA had the lowest at 49.15%.

R1.13: Line 240-251. Specify what S-EDQ1 means. It is the first time that the abbreviation has been used but I cannot find what it corresponds to in the text. In addition to mentioning it in the Abbreviations section, it should also be included in the main text.
Authors' response: In the edited document, the S-EDQ was first used on page 9, line 220 and the acronym has been fully specified.
Changes made: Page 9, line 220: We referred to these quintiles as Socio-economic deprivation quintiles (S-EDQ1 – S-EDQ5) in this paper.

R1.14: Again, I would suggest including additional analysis on inequality in vaccination coverage, for example, differences by urban and rural sector, between migrants, between indigenous and non-indigenous populations. Particularly, in the qualitative part, the differences between the urban and rural sectors are mentioned; It would be interesting to see if these differences are evident in the quantitative part.
Authors' response: We have included a section on rural and urban sector in the quantitative results section.
Change made: Refer to R1.11 above.

Discussion

R.1.15: Line 430. What does HIT mean?
Authors' response: HIT is an acronym for Herd Immunity Threshold. It was first mentioned in line 63. We have included the meaning of the acronym.
Change made: Page 3, line 61 – 63: Despite all the efforts to improve measles immunisation coverage, the proportion of immunised population with measles antigen containing vaccine (MCV) remains under the 95 percent herd immunity threshold (HIT).

R1.16: Line 433. To what period of time do these contextual adaptations refer (between 2002-2013? Or later).
Author's response: The contextual adaptations period spanned from 2002 to 2013, employed currently and in the future depending on needs.
Changes made: Page 20, lines 487 – 490: All these efforts are towards tailoring immunisation service to the targeted beneficiaries [63]. These approaches were employed in different combinations by RHAs in the past, currently and into the future with modifications depending on contexts.

R1.17: Since when did promoters of community programs begin to be introduced? Could it be observed if the presence of community program promoters really had an effect on vaccination coverage?
Authors' response: Community program promoters were first introduced in 2007 to address the inequities in vaccination coverage from the inner-city core neighborhoods. It was effective in improving the coverage even though it was capital intensive.
Change made: Page 20, lines 470 – 473: Particularly, regional health authorities where targeted approaches were introduced observed improvement in immunisation uptake by the initiation and maintenance of community program builders recruited from the community to reach out to the parents and clients in the inner-city core neighborhoods in Saskatoon RHA in 2007.
Page 20, lines 479 – 481: The above efforts are part of the strategies and interventions that increased uptake of measles immunisation especially by bridging the gap in the socio-economic divide with a resultant improvement in the coverage rates over the period under study.

R1.18: Line 448. What period of time do you refer to?
Authors' response: We referred to the targeted approach to immunization period which started in Saskatchewan in 2007
Changes made: Page 20, lines 482 – 486: During the targeted approach period starting from 2007, many regional health authorities in the province of Saskatchewan utilized reminder systems more effectively, increased the number of immunisation service delivery points and became more flexible in terms of clinic opening hours to allow access for caregivers who could not attend within the strict regular opening hours

R1.19: Line 454 and following. I suggest, for the reader's clarity, to make a summary table of the changes over time in health strategies and policies related to vaccination, which are mentioned both in the results and in the discussion.
Authors' response: The changes made varied in the respective RHAs. We have summarized the outcomes of the changes under the different themes in Fig 7 on page 19 of the manuscript.
Changes made: None

Figures.

R1.20: Figure 2. Include a note at the bottom of the figure that explains the graph and legends. Explain what S-EDQ is.
Author's response: The legend can be found on the figures.
Changes made: None

R1.21: Figure 5. Include a footnote to the figure that explains the graph and captions. Explain what is DEP 1, 2 etc.
Author's response: Footnote has been included. The DEP 1, 2 etc. are the same as S-EDQ 1, 2 etc. For uniformity and consistency, we have changed the DEP to S-EDQ on the graph.

Changes made: As indicated in the authors' response above.

R1.22: Figure 6. Include title, explain the main elements of the figure in a footnote.
Author's response: The title is indicated under the figure in the main document. The main elements were described in the main document. Figure 6 has become Figure 7 because of the inclusion of additional graph on rural and urban differences under results as part of the authors' response to R1.11.
Changes made: Page 18: lines 441 – 447: Figure 7 summarizes the results with various issues and clusters representing key building blocks—individual, social and institutional—that should be considered towards optimizing immunisation in the province. Each of these issues has critical implications for equity and public health policy. The framework includes individual, social and institutional dimensions of existing barriers and enablers and suggests a holistic, interconnected approach that integrates issues such as timeliness of measles immunisation dosages, population growth, urban-rural dichotomy, and regional differences and disparities in coverage rates.
