# Supplementary material for: Trends, barriers and enablers to measles immunisation coverage in Saskatchewan, Canada: A mixed methods study
Source: PLoS One. 2022 Nov 23;17(11):e0277876. doi: 10.1371/journal.pone.0277876 (PMC9683619; doi:10.1371/journal.pone.0277876)
Supplement: Attachment — Submitted filename: Response to Reviewers.docx [file pone.0277876.s003.docx]

Authors’ response: Trends, Barriers and Enablers to Measles Immunization Coverage in Saskatchewan, Canada: a Mixed Methods Study

Reviewer #1: General comment
R1.1: The topic of the article proposed by the researchers is interesting, it is a contribution to the analysis of measles vaccination trends, from a mixed approach. The data is limited to the province of Saskatchewan but could be understood given the qualitative approach the authors add to the study.
Authors response: Thank you for your constructive comments

Changes made: None

R1.2: However, I am concerned about the lack of concurrency in the temporality of the quantitative and qualitative approaches. I can understand that the quantitative data for this study only includes up to the year 2013, while the qualitative analysis would correspond to a more current information collection (the authors do not report on this). If so, this temporal gap would mean that the social, historical and cultural context in which the analysis is carried out is not the same. Ideally, I would suggest to the authors to extend the trend analysis to a more current date, closer to the period in which the data from the qualitative approach were collected.
Authors response: We looked more into contextuality in this study. The study looked at data from 2002 to 2013 which were the available data in the Saskatchewan Immunization registry at the time of the study. We intentionally used data to make the key informants (mostly experienced managers) aware of the gap and or challenges in measles immunization coverage to have them pursue vigorously quality and equity initiatives. The first interview with the key informants took place in the third quarter of 2016 at which time the only available data on the SIMS registry were updated until 2013 and the interviews were with people who had been working in the system for many years and they reflected over that history and brought to fore what they have done in that period. The information pertaining to their work in those periods when the data were collected were asked. The information acquired were aimed at informing future interventions and practice. We conducted the first interview when they had not seen the findings from the quantitative analysis. The coverage rates that the RHAs had seen before our study was the overall coverage for their respective RHAs as made available by the Ministry of Health in a non-granular form like our study did. Our study looked at rural-urban disparity in coverages as well as different socio-economic groups. We then collated the generalized themes to see what were done in different RHAs and compared that with the outcome in terms of coverage. Subsequently, we shared the results of the quantitative analysis with the same group of interviewees to see if they have made improvements or not especially with new evidence and the new details on geographical and socio-economic analysis. With that level of analysis, we carried out knowledge translation with the informants. This led to the second stage interviews in 2018, when we interrogated the same set of people about the past and reflect on how they wanted to use the new information to make future programmatic changes. We think their responses reflected not just that point in time but their experience over the last decade and their future plans, therefore, there is no substantial gap that could have affected the results.

Changes made: Page 7, line 168 – 174: Eighty-three percent (n=15) of the interview participants had at least eleven years’ experience, either in their present positions or in Saskatchewan RHAs in various public and population health capacities; with MHOs, an average of thirteen years’ experience and Coordinators/Front-line Immunization Officers, an average of twenty years. The information asked from the participants pertained to their work in those periods when the quantitative data were collected; hence they were reflecting on their experience during the time data were collected, taking into account the past and future implications.

Also, page 23, lines 563 – 567: However, the 2016 and 2017 qualitative data provided by key informants, majority of who were long serving in the RHAs were valuable in providing a comprehensive picture of trends and challenges faced in their respective RHAs addressing both interventions in the retrospective periods dating to a decade and how the new information will be used to make changes in the future.

Summary
R1.3: Line 32. It is not clear in what time or periods of time the interviews with the key informants were taken.

Authors response: We have added the time of the interview

Changes made: Page 2, Lines 30 – 33: We utilized the entire population of 16,582 children under two years of age available in the Saskatchewan Immunization Management System (SIMS) registry for 2002 and 2013 in aggregate format and interviewed 18 key informants in pre-determined two-stages in 2016 and 2017.

Introduction
R1.4: Lines 75-78. Specify in the province of Saskatchewan, what is the regular schedule of measles vaccination in the first and second doses.

Authors’ response: The routine schedule for first and second measles vaccination has been specified.

Change made: Page 3, lines 73 – 75: The Saskatchewan routine measles immunisation schedule are two doses of measles containing vaccine with first dose recommended at one year (12 months) and a second dose starting from 18 months of age.[1]

Materials and methods
R1.5: Line 145. Specify if the vaccination coverage was obtained: annually? For the entire province or for each of the regions?

Authors’ response: As recommended, we have added “annualized”

Changes made: Page 6, line 151 – 152: We used annualized data from two population groups, aged 12-14 months and 18-24 months.

R1.6: Justify why a data analysis is performed only up to 2013, why more recent data is not included.
Authors’ response: This study took place at a period when health system transformation was taking place hence, we used all the available data up to that point.

Change made: Page 6, lines 145 – 149: This study took place at a period when health system transformation was taking place (i.e., from Saskatchewan Immunization Registry to the Panorama Gateway). The only available data at the time of study were up to 2013, hence the study used the total sample available in the province of Saskatchewan Immunization Management Registry.

R1.7: Lines 153-156. Describe in what time period the key informant interviews were conducted.

Authors’ response: The time period has been specified.

Change made: Page 7, lines 159 – 164: All the selected eighteen key informants participated in the first phase of interviews while seventeen participated in the second phase. The first stage of interviews which took place in the fourth quarter of 2016 collected the interviewees’ initial general perspectives on factors that promote or hinder measles immunisation uptake in their health regions while the second stage, in the fourth quarter of 2017, was conducted to validate key themes and insights from the initial interviews and after quantitative results were presented to the participants

R1.8: Describe whether there was triangulation of qualitative information and how it was done.

Authors’ response: To ensure consistency in information received from each of the health regions, we interviewed two key informants.

Change made: Page 7, line 165 – 166: Qualitative data triangulation was ensured by interviewing two stakeholders each from each regional health authority.

R1.9: How do the authors justify a quantitative analysis of data from 2010-2013, while the qualitative analysis corresponds to a current period of time? That is, although there is a mixed approach to the research, the authors have worked with a quantitative and qualitative approach at two different, non-concurrent moments.

Authors’ response: See response above (R1.2)

Change made: Response R1.2 above

Statistical analysis
R1.10: An analysis of differences by socioeconomic quintiles is carried out in the results. It is necessary to describe in the Statistical analysis section where this information was obtained and how it was included in the analysis.

Authors’ response: We have described how socioeconomic quintiles were estimated in the methods section

Changes made: Page 9, lines 212 – 221: To assess socio-economic disparities, we used the index of deprivation developed at the Institut National de Sante Publique du Quebec (INSPQ) which measures deprivation at the level of dissemination areas (DAs), the smallest areas for which Census data are available in Canada, comprising of approximately 400 to 700 residents living in the same small area geography[50]. The deprivation index looked at social and material dimensions with the proportion of single parents, the proportion of residents living alone, and marital status in the social components while the material deprivation measured educational attainment, average income, and employment status variables.[50] The DAs were divided into five quintiles 1 to 5 (Q1 to Q5) where each quintile represented 20% of population with Q1 being the most privileged and Q5, the least. We referred to these quintiles as Socio-economic deprivation quintiles (S-EDQ1 – S-EDQ5) in this paper.

R1.11: I consider that, when evaluating the disparity in measles coverage by socioeconomic status, the authors could delve deeper into the results, there are some inequalities analyzes that could perhaps be applied in the present study, as well as exploring differences by urban, rural, between migrants, between indigenous and non-indigenous populations
Authors’ response: Your suggestions are well noted. We have included information on urban and rural analysis in the results section as well as in the discussion. However, immunization data by ethnicity and immigration status were not systematically collected in the province of Saskatchewan. Also, the authors did not have access to individual but aggregated data for the study. We have hence noted this as part of study limitations.

Changes made: Pages 11 – 12, lines 268 – 278:

Assessing disparity in measles coverage by geographical location

There was increase in the coverage trends in Saskatchewan rural and urban locations with coverage rates being higher in the rural than in the urban locations. The coverage rate among on-time for second dose at 2-year (MCV2) increased between 2002 and 2008 (APC: 0.8, 95%CI: ), followed by a higher progressive increase from 2008 till 2013 (APC: 3.0, 95%CI: ) for the rural locations. . The urban location coverage rates displayed an increase from 2002 – 2008 at an APC of 2.3, a further increase of APC of 4.5 from 2008 – 2011 with the rate of change being significant with CIs of 1.7 – 3.0 and 1.6 – 7.5 respectively while the increase from 2011 – 2013 and an APC of 2.0, was not significant (CI -0.4 – 4.6). A test for parallelism of the two coverage rate trends for the on-time for MCV2 in the urban and rural locations to check for any difference between the two trend lines indicated no parallelism (p-value = 0.027) indicating a significant difference between the trend lines of the two geographical locations.

Page 13, lines 301 – 305: Summarizing, there was progressive increase in the coverage rates for both S-EDQ 1 and 5 and among both rural and urban populations for MCV2 in the province of Saskatchewan. The coverage rates were higher in the S-EDQ1 than S-EDQ5 and among the rural dwellers than the urban inhabitants among the on-time for MCV2 all through 2002 – 2013.

Page 24, line 568 – 570: It should be noted that data were not available for ethnicity and immigration status. Future studies are needed to explore patterns of measles immunization among other sub-populations which frequently exhibit inequities such as immigrants and indigenous population.

Results

R1.12: Lines 223-224. Are you referring to first or second dose coverage for measles, or full schedule coverage?
Authors’ response: We have included that second dose for measles (MCV2) was used which is also the full schedule according to World Health Organisation (WHO).

Change made: Page 11, line 250 – 251: From MCV2 results for 2002, Sun Country RHA had the highest coverage of 67.22% while Prairie North RHA had the lowest at 49.15%.

R1.13: Line 240-251. Specify what S-EDQ1 means. It is the first time that the abbreviation has been used but I cannot find what it corresponds to in the text. In addition to mentioning it in the Abbreviations section, it should also be included in the main text.
Authors’ response: In the edited document, the S-EDQ was first used on page 9, line 220 and the acronym has been fully specified.

Changes made: Page 9, line 220: We referred to these quintiles as Socio-economic deprivation quintiles (S-EDQ1 – S-EDQ5) in this paper.

R1.14: Again, I would suggest including additional analysis on inequality in vaccination coverage, for example, differences by urban and rural sector, between migrants, between indigenous and non-indigenous populations. Particularly, in the qualitative part, the differences between the urban and rural sectors are mentioned; It would be interesting to see if these differences are evident in the quantitative part.

Authors’ response: We have included a section on rural and urban sector in the quantitative results section.

Change made: Refer to R1.11 above.

Discussion

R.1.15: Line 430. What does HIT mean?

Authors’ response: HIT is an acronym for Herd Immunity Threshold. It was first mentioned in line 63. We have included the meaning of the acronym.

Change made: Page 3, line 61 – 63: Despite all the efforts to improve measles immunisation coverage, the proportion of immunised population with measles antigen containing vaccine (MCV) remains under the 95 percent herd immunity threshold (HIT).

R1.16: Line 433. To what period of time do these contextual adaptations refer (between 2002-2013? Or later).

Author’s response: The contextual adaptations period spanned from 2002 to 2013, employed currently and in the future depending on needs.

Changes made: Page 20, lines 487 – 490: All these efforts are towards tailoring immunisation service to the targeted beneficiaries [63]. These approaches were employed in different combinations by RHAs in the past, currently and into the future with modifications depending on contexts.

R1.17: Since when did promoters of community programs begin to be introduced? Could it be observed if the presence of community program promoters really had an effect on vaccination coverage?

Authors’ response: Community program promoters were first introduced in 2007 to address the inequities in vaccination coverage from the inner-city core neighborhoods. It was effective in improving the coverage even though it was capital intensive.

Change made: Page 20, lines 470 – 473: Particularly, regional health authorities where targeted approaches were introduced observed improvement in immunisation uptake by the initiation and maintenance of community program builders recruited from the community to reach out to the parents and clients in the inner-city core neighborhoods in Saskatoon RHA in 2007.

Page 20, lines 479 – 481: The above efforts are part of the strategies and interventions that increased uptake of measles immunisation especially by bridging the gap in the socio-economic divide with a resultant improvement in the coverage rates over the period under study.

R1.18: Line 448. What period of time do you refer to?

Authors’ response: We referred to the targeted approach to immunization period which started in Saskatchewan in 2007

Changes made: Page 20, lines 482 – 486: During the targeted approach period starting from 2007, many regional health authorities in the province of Saskatchewan utilized reminder systems more effectively, increased the number of immunisation service delivery points and became more flexible in terms of clinic opening hours to allow access for caregivers who could not attend within the strict regular opening hours

R1.19: Line 454 and following. I suggest, for the reader's clarity, to make a summary table of the changes over time in health strategies and policies related to vaccination, which are mentioned both in the results and in the discussion.

Authors’ response: The changes made varied in the respective RHAs. We have summarized the outcomes of the changes under the different themes in Fig 7 on page 19 of the manuscript.

Changes made: None

Figures.

R1.20: Figure 2. Include a note at the bottom of the figure that explains the graph and legends. Explain what S-EDQ is.

Author’s response: The legend can be found on the figures.

Changes made: None

R1.21: Figure 5. Include a footnote to the figure that explains the graph and captions. Explain what is DEP 1, 2 etc.

Author’s response: Footnote has been included. The DEP 1, 2 etc. are the same as S-EDQ 1, 2 etc. For uniformity and consistency, we have changed the DEP to S-EDQ on the graph.

Changes made: As indicated in the authors’ response above.

R1.22: Figure 6. Include title, explain the main elements of the figure in a footnote.

Author’s response: The title is indicated under the figure in the main document. The main elements were described in the main document. Figure 6 has become Figure 7 because of the inclusion of additional graph on rural and urban differences under results as part of the authors’ response to R1.11.

Changes made: Page 18: lines 441 – 447: Figure 7 summarizes the results with various issues and clusters representing key building blocks—individual, social and institutional—that should be considered towards optimizing immunisation in the province. Each of these issues has critical implications for equity and public health policy. The framework includes individual, social and institutional dimensions of existing barriers and enablers and suggests a holistic, interconnected approach that integrates issues such as timeliness of measles immunisation dosages, population growth, urban-rural dichotomy, and regional differences and disparities in coverage rates.
